# Synchronous Metaplastic Breast Carcinoma with Mesenchymal Differentiation and Triple-Negative Invasive Papillary Carcinoma: A Case Report and Literature Review

**DOI:** 10.3390/jcm15197705

**Published:** 2026-10-05

**Authors:** Vladimir Tvrdisic, Vesna Vulovic, Bojan Stojanovic, Marina Petronijevic, Milica Dimitrijevic, Emilija Subanovic, Nina Radosavljevic, Marko Spasic, Bojan Milosevic, Jasna Gacic

**Affiliations:** 1 Clinic for General Surgery, University Clinical Center Kragujevac, 34000 Kragujevac, Serbia; 2 Department of Surgery, University Hospital Medical Center Bežanijska Kosa, 11000 Belgrade, Serbia; 3 Department of Surgery, Faculty of Medical Sciences, University of Kragujevac, 34000 Kragujevac, Serbia; 4 Center for Molecular Medicine and Stem Cell Research, Faculty of Medical Sciences, University of Kragujevac, 34000 Kragujevac, Serbia; 5 Center for Medical Oncology, University Clinical Center Kragujevac, 34000 Kragujevac, Serbia; 6 Center for Plastic, Reconstructive and Aesthetic Surgery, University Clinical Center Kragujevac, 34000 Kragujevac, Serbia; 7 Center for Radiation Oncology, University Clinical Center Kragujevac, 34000 Kragujevac, Serbia; 8 Department of Surgery with Anesthesiology, Faculty of Medicine, University of Belgrade, 11000 Belgrade, Serbia

**Keywords:** metaplastic breast carcinoma, invasive papillary carcinoma, synchronous breast tumors, triple-negative breast cancer, sampling error, lymph node metastasis, PD-L1, pembrolizumab

## Abstract

**Background:** Metaplastic breast carcinoma with mesenchymal differentiation is a rare and morphologically heterogeneous malignancy that may show a biphasic epithelial and sarcomatoid pattern historically termed “carcinosarcoma.” Its synchronous occurrence with invasive papillary carcinoma is exceptionally uncommon and may create diagnostic and therapeutic difficulties. **Methods:** We report the clinicopathological course of a premenopausal woman with two synchronous breast malignancies. Clinical, radiological, histopathological, and immunohistochemical findings were reviewed, including PD-L1 assessment using the 22C3 antibody, and interpreted in the context of the relevant literature. **Results:** Definitive surgical pathology demonstrated a 125-mm metaplastic breast carcinoma with mesenchymal differentiation showing carcinosarcoma morphology and a separate 22-mm high-grade triple-negative invasive papillary carcinoma. Sarcomatoid predominance led to the initial interpretation of malignant phyllodes tumor on core biopsy. Axillary lymph-node metastases showed papillary morphology and a concordant immunophenotype, strongly supporting their relationship to the invasive papillary carcinoma. Later supraclavicular recurrence and pulmonary metastases could not be conclusively attributed to either primary tumor. PD-L1 combined positive scores were 25 in both primary tumors and 35 in the supraclavicular metastasis. Pembrolizumab-based therapy was followed by radiological regression and clinical stability. **Conclusions:** This case highlights the importance of representative sampling, careful characterization of synchronous tumors, comparison of metastatic deposits with all primary lesions, and biomarker reassessment during progression. The observed response should be interpreted in the context of PD-L1-positive metastatic triple-negative breast cancer rather than as evidence of histology-specific pembrolizumab efficacy.

## 1. Introduction

Most primary malignant tumors of the breast are epithelial carcinomas arising from structures of the terminal duct–lobular unit [1]. Invasive breast carcinoma of no special type represents the most frequent histological form, whereas metaplastic breast carcinoma constitutes a rare and morphologically heterogeneous group of malignancies, accounting for less than 1% of breast cancers [2,3]. Metaplastic carcinomas are characterized by differentiation of neoplastic epithelial cells toward nonglandular epithelial or mesenchymal-appearing elements and generally exhibit more aggressive biological behavior than conventional invasive breast carcinomas [3,4].

A distinctive but uncommon pattern of metaplastic breast carcinoma is the coexistence of malignant epithelial and sarcomatoid components within the same tumor. This biphasic morphology was historically described as breast carcinosarcoma, although it is now regarded as part of metaplastic breast carcinoma rather than as a separate diagnostic entity [5]. The relative proportions and spatial distribution of these components may vary considerably, producing marked intratumoral heterogeneity [6]. Consequently, recognition of the epithelial component may be difficult when the sarcomatous component predominates, particularly in limited core needle biopsy specimens [7]. In addition, tumors historically described as breast carcinosarcoma frequently exhibit a triple-negative phenotype, limiting the applicability of endocrine and human epidermal growth factor receptor 2 (HER2)-targeted therapies [8].

We report a premenopausal woman with two synchronous breast malignancies: a metaplastic breast carcinoma with mesenchymal differentiation showing carcinosarcoma morphology and a spatially separate high-grade triple-negative invasive papillary carcinoma. The metaplastic carcinoma was dominated by a pleomorphic mesenchymal component and was initially interpreted as a malignant phyllodes tumor on core biopsy. Definitive surgical pathology established the presence of both primary tumors and also revealed axillary lymph-node metastases. The morphology and immunophenotype of these nodal deposits favored the smaller invasive papillary carcinoma as their source. During follow-up, the patient developed supraclavicular nodal recurrence and pulmonary metastases. Unlike the initial axillary metastases, however, these later lesions could not be confidently attributed to either primary tumor on the basis of the available pathological and imaging findings, and comparative molecular profiling was not performed to clarify their clonal origin. PD-L1 testing of the recurrent disease identified a therapeutically relevant biomarker, and pembrolizumab-based treatment was followed by radiological regression and sustained clinical stability. The case therefore illustrates the diagnostic difficulties created by marked intratumoral heterogeneity, synchronous histologically distinct breast cancers, and uncertainty regarding the origin of subsequent metastatic disease, while also highlighting the potential clinical value of biomarker-guided systemic therapy. This case report was prepared in accordance with the CARE (CAse REport) reporting guidelines [9].

## 2. Case Report

### 2.1. Patient History and Initial Clinical Presentation

A 40-year-old premenopausal woman presented with a progressively enlarging mass in the right breast, first noticed during breastfeeding in early 2022, together with an enlarged right axillary lymph node. She had no significant medical history, was not receiving regular medication, and had no family history of breast cancer or other relevant hereditary malignancies.

**Figure 1:**
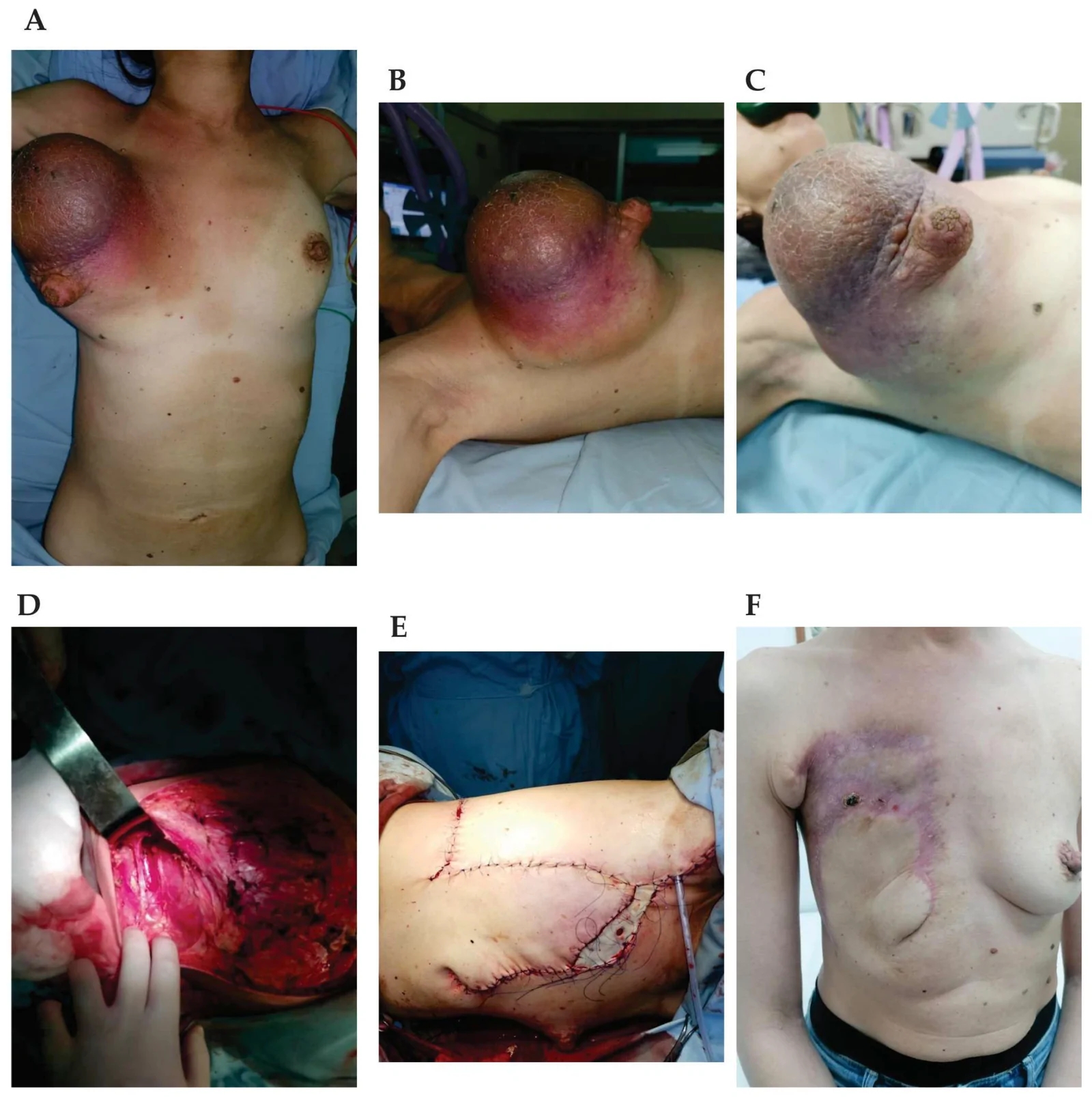
Clinical presentation, surgical treatment, and postoperative outcome. (**A**) Preoperative frontal view showing marked enlargement of the right breast caused by the large tumor, with tense erythematous skin and prominent superficial vascularity. (**B**) Oblique preoperative view demonstrating the extensive tumor mass occupying almost the entire right breast and producing marked distortion of the breast contour. (**C**) Closer oblique view showing pronounced skin stretching and discoloration with distortion and displacement of the nipple–areolar complex. (**D**) Intraoperative appearance of the extensive skin and soft-tissue defect following right modified radical mastectomy. (**E**) Immediate postoperative appearance after reconstruction using a transposition skin flap and a free full-thickness skin graft according to Wolfe. (**F**) Follow-up appearance of the reconstructed right chest wall showing satisfactory integration of the flap and skin graft with mature postoperative scarring.

On clinical examination on 14 July 2022, the right breast was almost entirely occupied by a firm, non-tender mass measuring approximately 10 cm and involving the central and both upper quadrants. The affected breast was markedly enlarged, with pronounced distortion of its normal contour. The overlying skin was tense, erythematous, and fixed to the underlying tumor, with visible superficial vascularity and distortion of the nipple–areolar complex (Figure 1A–C). A conglomerate of clinically abnormal lymph nodes measuring up to approximately 4 cm was palpable in the lower right axilla. Clinical examination of the contralateral breast and axilla revealed no pathological findings.

### 2.2. Diagnostic Work-Up and Preoperative Assessment

Ultrasonography of the breasts and regional lymphatic basins demonstrated a hypoechoic, vertically oriented tumor involving the central, upper outer, and upper inner quadrants of the right breast. The lesion showed increased internal vascularity on color Doppler examination and extended to the overlying skin, with a clearly visualized line of fixation. A conglomerate of cystic lesions measuring up to 18 mm was identified near the junction of the upper quadrants toward the sternum. In the lower right axilla, a conglomerate of morphologically abnormal lymph nodes measured 31 mm in aggregate, with the largest individual lymph node measuring 18 mm. Two additional pathological lymph nodes were identified in the right infraclavicular region, the larger measuring 13 × 10 mm. No focal lesions were detected in the left breast, and the left axilla was sonographically unremarkable. The findings were classified as Breast Imaging Reporting and Data System category 5 in the right breast and category 2 in the left breast.

The patient was first evaluated by the institutional multidisciplinary breast team on 27 July 2022. An ultrasound-guided core needle biopsy of the right breast mass and urgent contrast-enhanced computed tomography of the thorax and abdomen were recommended. Core needle biopsy was performed on 9 August 2022. Histopathological examination demonstrated a malignant proliferation composed of markedly pleomorphic cells, some with a bizarre appearance and prominent nucleoli. The tumor cells expressed vimentin but lacked pancytokeratin AE1/AE3, progesterone receptor, and HER2 expression. The Ki-67 proliferation index was heterogeneously distributed and averaged approximately 25%. Based on the limited biopsy sample and the predominantly mesenchymal morphology and immunophenotype, the lesion was interpreted as a malignant mesenchymal proliferation, favoring malignant phyllodes tumor. Definitive distinction from a predominantly sarcomatous metaplastic carcinoma was not possible on the core biopsy specimen.

**Figure 2:**
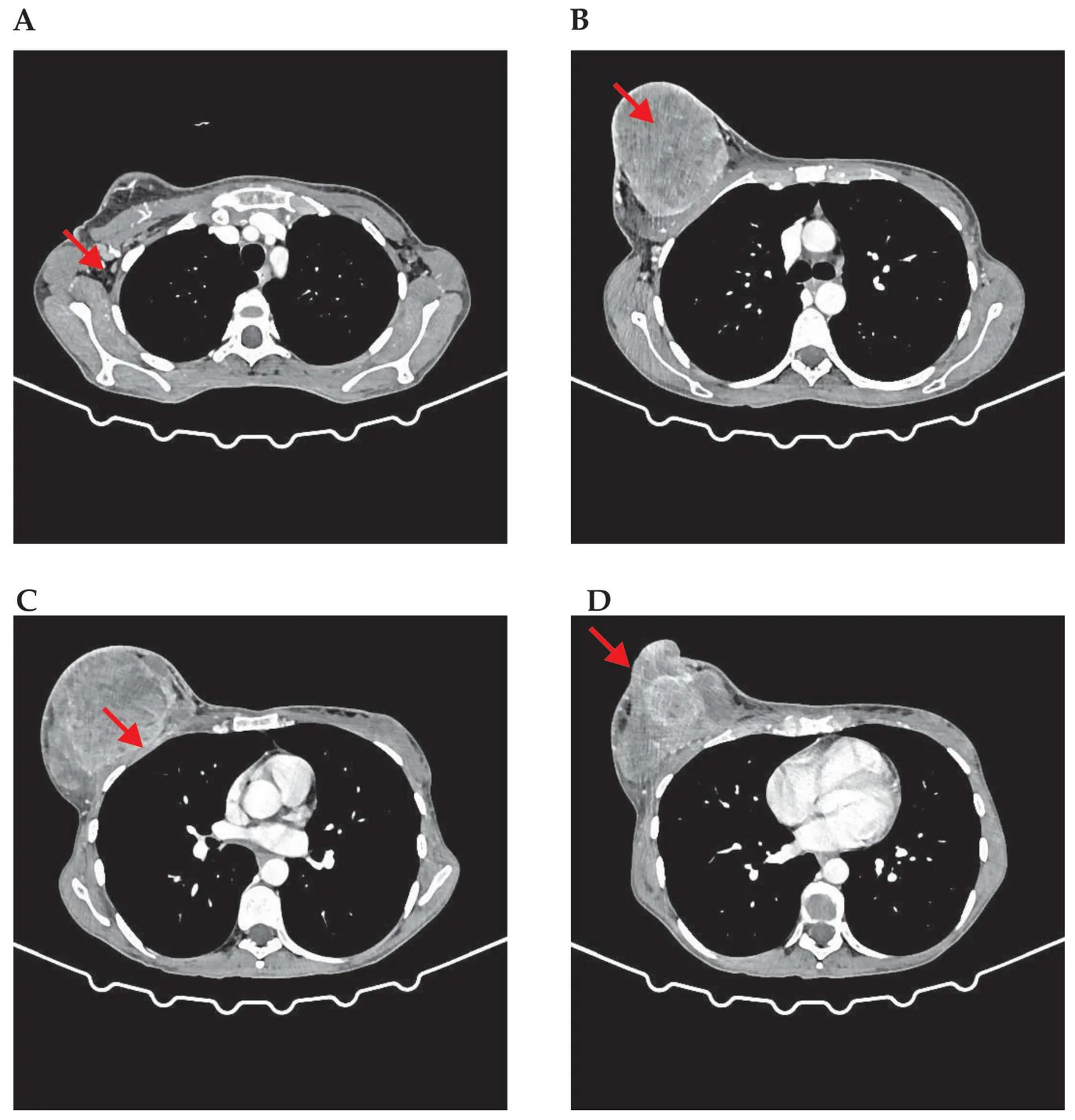
Preoperative contrast-enhanced computed tomography findings. (**A**) Axial contrast-enhanced computed tomography (CT) image at the level of the upper thorax showing pathological right axillary and infraclavicular lymph nodes (red arrow). (**B**) More caudal axial CT section showing the large, relatively well-demarcated heterogeneous right breast mass occupying most of the breast (red arrow). (**C**) Axial CT image at the level of the main pulmonary arteries showing heterogeneous internal enhancement of the tumor and broad posterior contact with the pectoral fascia; the red arrow indicates the posterior tumor margin. (**D**) More caudal axial CT section showing the inferior and anterior extent of the tumor; the red arrow indicates involvement of the nipple–areolar region. No definite intrathoracic metastatic lesions were identified on the preoperative examination.

Contrast-enhanced computed tomography performed on 17 August 2022 demonstrated pathological right axillary and infraclavicular lymph nodes (Figure 2A) and a large, relatively well-demarcated, heterogeneous tumor occupying almost the entire right breast (Figure 2B). Posteriorly, the lesion showed broad contact with the pectoral fascia, although no unequivocal radiological evidence of pectoral muscle invasion was identified (Figure 2C). More caudally, the tumor extended anteriorly toward and involved the nipple–areolar region (Figure 2D). Several smaller cyst-like lesions were present adjacent to the dominant tumor. Several heterogeneous pathological lymph nodes were identified in the right axilla, measuring up to 15 × 11 mm. A small parenchymal cyst was noted in the liver, while no definite radiological evidence of distant metastatic disease was detected in the thorax or abdomen. At the second multidisciplinary breast-team evaluation, the patient had an Eastern Cooperative Oncology Group performance status of 0, and definitive surgical treatment was recommended.

### 2.3. Surgical Treatment and Postoperative Course

On 1 September 2022, the patient underwent a right modified radical mastectomy according to Madden. Resection resulted in an extensive skin and soft-tissue defect of the right anterior chest wall (Figure 1D). Reconstruction was performed using a transposition skin flap and a free full-thickness skin graft according to Wolfe (Figure 1E). The immediate postoperative course was uncomplicated, and the surgical drain was removed on the fourth postoperative day.

At the initial plastic-surgery assessment, the skin graft had been accepted and showed early signs of revascularization. The distal portion of the transposition flap was livid, indicating compromised perfusion, although the surgical wounds remained dry and clean and were healing primarily. The patient was subsequently evaluated by a multidisciplinary team comprising specialists in plastic surgery, dermatovenereology, and orthopedic surgery and traumatology, who recommended 15 sessions of hyperbaric oxygen therapy.

At the follow-up examination on 29 September 2022, the flap was viable and the skin graft had been completely accepted. A limited area of dry necrosis measuring approximately 12 mm in width and 45 mm in length remained at the distal flap margin adjacent to the graft. Conservative local wound care was continued, without forced removal of the dry eschar. Subsequent follow-up demonstrated satisfactory integration of the flap and skin graft, with mature postoperative scarring of the reconstructed right chest wall (Figure 1F).

### 2.4. Histopathological and Immunohistochemical Findings

**Figure 3:**
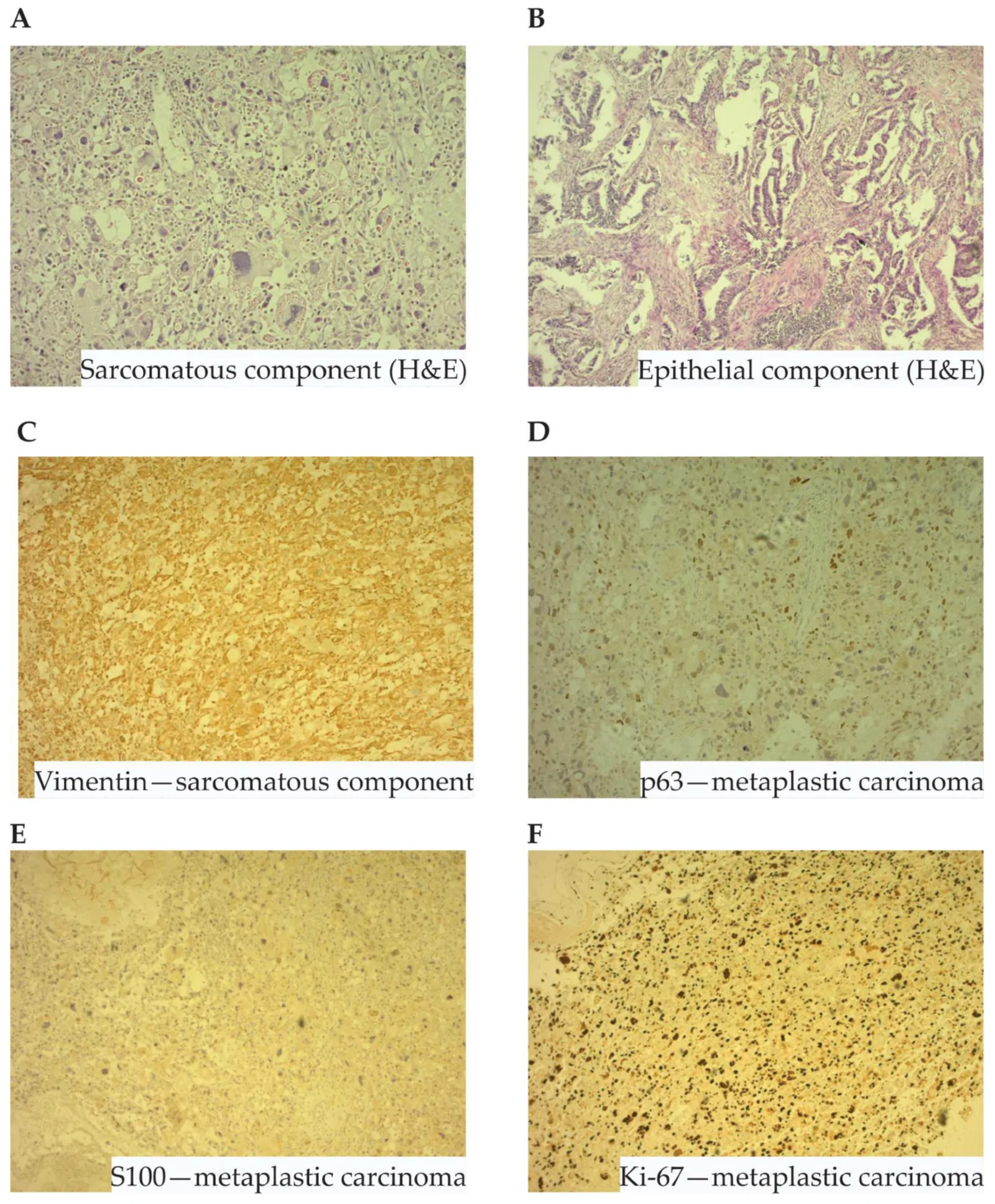
Histopathological and immunohistochemical features of metaplastic breast carcinoma with mesenchymal differentiation showing carcinosarcoma morphology. (**A**) Sarcomatoid component composed of highly pleomorphic malignant cells, including bizarre and multinucleated tumor giant cells (hematoxylin and eosin [H&E], original magnification ×100). (**B**) Malignant epithelial component consisting of irregular epithelial structures embedded within the tumor stroma (H&E, original magnification ×100). (**C**) Diffuse cytoplasmic vimentin immunoreactivity in the sarcomatoid component, supporting mesenchymal differentiation (vimentin immunohistochemistry, original magnification ×100). (**D**) Focal nuclear p63 immunoreactivity in tumor cells (p63 immunohistochemistry, original magnification ×100). (**E**) S100 protein immunoreactivity in tumor cells (S100 immunohistochemistry, original magnification ×100). (**F**) High and heterogeneous proliferative activity demonstrated by Ki-67 immunostaining (Ki-67 immunohistochemistry, original magnification ×100).

Gross and histopathological examination of the mastectomy specimen revealed two malignant tumors measuring 125 mm and 22 mm in greatest diameter, respectively. Both lesions were predominantly circumscribed. According to contemporary WHO terminology, the larger tumor was classified as metaplastic breast carcinoma with mesenchymal differentiation, showing carcinosarcoma morphology, and was composed of approximately 95% sarcomatoid/mesenchymal and 5% epithelial components. The sarcomatous component consisted of highly pleomorphic and frequently bizarre malignant cells, including numerous multinucleated tumor giant cells (Figure 3A). The epithelial component was represented by irregular malignant epithelial structures showing poor ductal differentiation (Figure 3B). Extensive areas of tumor necrosis and hemorrhage were present.

Immunohistochemically, the dominant tumor showed prominent mesenchymal differentiation, with diffuse vimentin expression in the sarcomatous component (Figure 3C), whereas focal pancytokeratin AE1/AE3 and p63 expression supported the presence of epithelial differentiation; representative focal nuclear p63 immunoreactivity is shown in Figure 3D. Additional immunoreactivity for S100 calcium-binding protein (S100) protein (Figure 3E), cluster of differentiation 68 (CD68), and smooth-muscle actin was documented. Estrogen receptor, HER2, cytokeratin 7 (CK7), and epithelial membrane antigen were not expressed. The Ki-67 proliferation index was heterogeneous and high, averaging approximately 60% (Figure 3F). The integrated pathological assessment classified the tumor within the triple-negative group.

**Figure 4:**
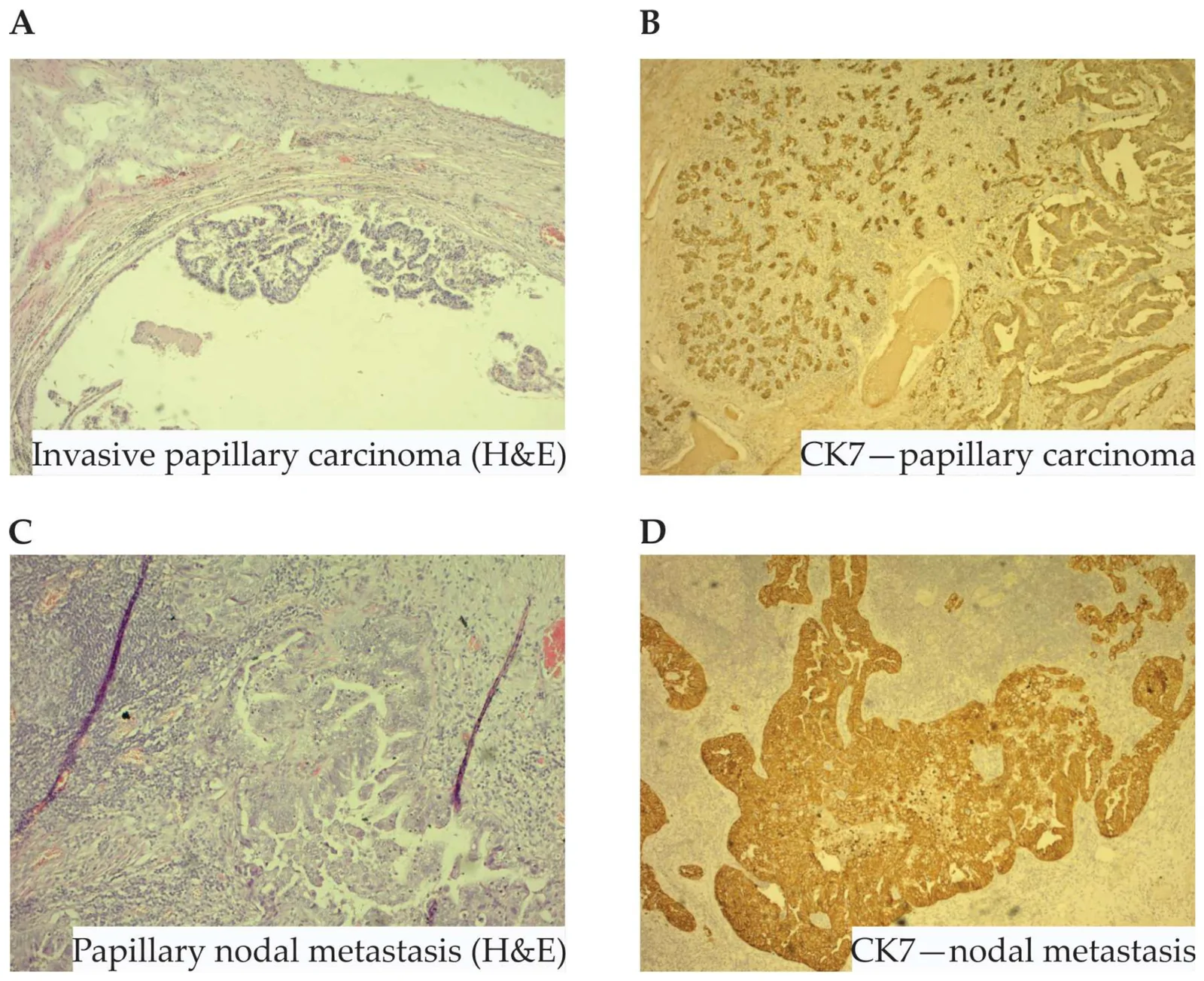
Histopathological and immunohistochemical features of invasive papillary carcinoma and the corresponding axillary lymph-node metastasis. (**A**) Representative hematoxylin and eosin section of the invasive papillary carcinoma showing true papillary structures with fibrovascular stromal cores and invasion into the surrounding stroma. Blue arrows indicate representative papillary structures with fibrovascular cores, whereas red arrows indicate areas of stromal invasion (H&E, original magnification ×40). (**B**) Cytokeratin 7 (CK7) immunoreactivity in the epithelial cells of the invasive papillary carcinoma (CK7 immunohistochemistry, original magnification ×40). (**C**) Axillary lymph-node metastasis reproducing papillary architecture with fibrovascular stromal cores (H&E, original magnification ×40). (**D**) CK7 immunoreactivity in the metastatic epithelial tumor cells, supporting the relationship of the nodal deposit to the invasive papillary carcinoma (CK7 immunohistochemistry, original magnification ×40).

The second tumor was a 22-mm invasive papillary carcinoma in which more than 90% of the invasive component showed true papillary architecture, meeting the current WHO morphological criteria for invasive papillary carcinoma. The invasive component consisted of complex papillary structures supported by well-developed fibrovascular stromal cores and lined by pleomorphic cuboidal epithelial cells with eosinophilic cytoplasm and vesicular nuclei (Figure 4A). At the tumor–stroma interface, irregular papillary and epithelial structures infiltrated the surrounding desmoplastic stroma, confirming genuine stromal invasion rather than a purely circumscribed papillary proliferation. The desmoplastic response was mild to moderate, and the stromal mononuclear inflammatory infiltrate was sparse. Extensive necrotic and hemorrhagic areas were also present. Immunohistochemically, the papillary carcinoma expressed CK7 (Figure 4B) and cytokeratin 5/6 (CK5/6) and lacked estrogen receptor, progesterone receptor, and HER2 expression. Its Ki-67 proliferation index was heterogeneously high and averaged approximately 40%.

Definite perineural, lymphatic, and vascular invasion were identified. A total of 14 axillary lymph nodes were examined. Metastatic carcinoma was present in two lymph nodes, while the remaining 12 showed chronic reactive lymphadenitis. Importantly, the nodal metastases demonstrated papillary differentiation (Figure 4C) and retained CK7 expression (Figure 4D), supporting their relationship to the invasive papillary carcinoma rather than to the predominantly sarcomatoid component of the metaplastic breast carcinoma. The metastatic deposits were cytokeratin 20 (CK20)-negative and lacked estrogen receptor, progesterone receptor, and HER2 expression, while the Ki-67 proliferation index averaged approximately 50%.

Based on the combined morphological and immunohistochemical findings, the final diagnosis was synchronous metaplastic breast carcinoma with mesenchymal differentiation showing carcinosarcoma morphology and invasive papillary carcinoma. The two macroscopically distinct invasive tumors were characterized separately: the metaplastic carcinoma measured 125 mm, whereas the invasive papillary carcinoma measured 22 mm. According to the AJCC staging approach for multiple synchronous invasive carcinomas within the same breast, the pathological T category is assigned according to the largest invasive tumor, with the suffix “(m)” indicating multiple macroscopically distinct invasive tumors. Accordingly, the overall pathological T category was pT3(m). Two of 14 axillary lymph nodes contained metastatic carcinoma. Their papillary architecture and concordant immunophenotypic profile strongly supported their origin from the invasive papillary carcinoma. However, the regional nodal category was recorded for the overall ipsilateral breast cancer presentation as pN1 rather than as a separate tumor-specific pN category. Both primary tumors were histological grade 3. Lymphatic and vascular invasion were present, and surgical margins were microscopically negative (R0). The final pathological classification was therefore recorded as pT3(m) pN1, G3, L1, V1, R0.

### 2.5. Adjuvant Treatment and Initial Surveillance

At the postoperative multidisciplinary breast-team meeting on 23 September 2022, adjuvant systemic chemotherapy and postoperative radiotherapy were recommended because of the large tumor size, high histological grade, nodal involvement, triple-negative phenotype, and metaplastic histology.

The patient received four cycles of doxorubicin and cyclophosphamide, followed by 12 weekly administrations of paclitaxel. Treatment was completed without documented severe hematological or nonhematological toxicity. Adjuvant radiotherapy was subsequently delivered to the right chest wall and ipsilateral regional lymphatic basins. According to the available radiotherapy plan, total doses of 48 Gy in 24 fractions and 50 Gy in 25 fractions were prescribed to the designated planning target volumes using 6-MV photon irradiation.

The patient continued regular oncological surveillance after completion of multimodal treatment. Follow-up computed tomography of the thorax and abdomen performed in September 2023 showed no evidence of locoregional recurrence or distant metastatic disease.

### 2.6. Recurrence and Metastatic Progression

In September 2025, approximately three years after surgery, ultrasonography of the neck demonstrated pathological lymph nodes in the right supraclavicular region. Two nodes measured approximately 8 and 6 mm, while two additional laterally located lesions were in close contact and had a combined diameter of approximately 15 mm. Ultrasound-guided core needle biopsy of a right supraclavicular lymph node was performed on 24 September 2025. Three tissue cores were obtained using a 16-gauge needle, without immediate procedural complications.

Histopathological examination demonstrated lymph-node tissue infiltrated by carcinoma arranged in solid formations. The tumor cells were polygonal, with eosinophilic cytoplasm, enlarged hyperchromatic to vesicular nuclei, variable nuclear pleomorphism, and prominent nucleoli. Immunohistochemically, the metastatic cells expressed E-cadherin and lacked estrogen receptor, progesterone receptor, and HER2 expression. The Ki-67 proliferation index was heterogeneous and averaged approximately 40%. The findings were consistent with moderately differentiated breast carcinoma of no special type metastatic to a supraclavicular lymph node, with a triple-negative phenotype. Because the recurrent carcinoma lacked the previously documented papillary architecture and its available morphological and immunophenotypic features were not specific for either synchronous primary malignancy, its exact relationship to the invasive papillary carcinoma or the metaplastic carcinoma could not be established.

Staging computed tomography of the thorax, abdomen, and pelvis performed in October 2025 demonstrated multiple pulmonary metastases, confirming progression to metastatic disease. The pulmonary lesions were identified radiologically and were not biopsied; consequently, their histological origin could not be determined. Programmed death-ligand 1 (PD-L1) expression was subsequently assessed using the 22C3 antibody in representative tissue from both synchronous primary breast tumors and from the supraclavicular nodal metastasis. The metaplastic breast carcinoma with mesenchymal differentiation and the invasive papillary carcinoma each showed a combined positive score (CPS) of 25, whereas the supraclavicular nodal metastasis showed a CPS of 35. All evaluated specimens were adequate for interpretation, and PD-L1-positive immune cells were present in each specimen.

### 2.7. Treatment of Metastatic Disease and Current Outcome

Given the triple-negative phenotype and a PD-L1 combined positive score of 35 in the biopsied supraclavicular recurrence, pembrolizumab-based systemic therapy was initiated on 29 January 2026. Pembrolizumab was administered together with paclitaxel and carboplatin at the treating oncology center. Paclitaxel was subsequently discontinued because of a hypersensitivity reaction, while carboplatin was continued without paclitaxel. The available records document 12 administrations of carboplatin. Pembrolizumab was subsequently continued as maintenance treatment.

The patient maintained an Eastern Cooperative Oncology Group performance status of 0 throughout treatment. During systemic therapy, transient elevations in serum aminotransferases were documented, with aspartate aminotransferase of 84 U/L and alanine aminotransferase of 127 U/L. Viral screening was negative. No clinical deterioration or interruption of pembrolizumab because of immune-related toxicity was documented.

Follow-up multidetector computed tomography of the neck, thorax, and abdomen performed on 14 May 2026, demonstrated regression of the previously documented lesions, while the overall oncological assessment classified the disease as stable. The multidisciplinary team therefore recommended continuation of pembrolizumab.

**Figure 5:**
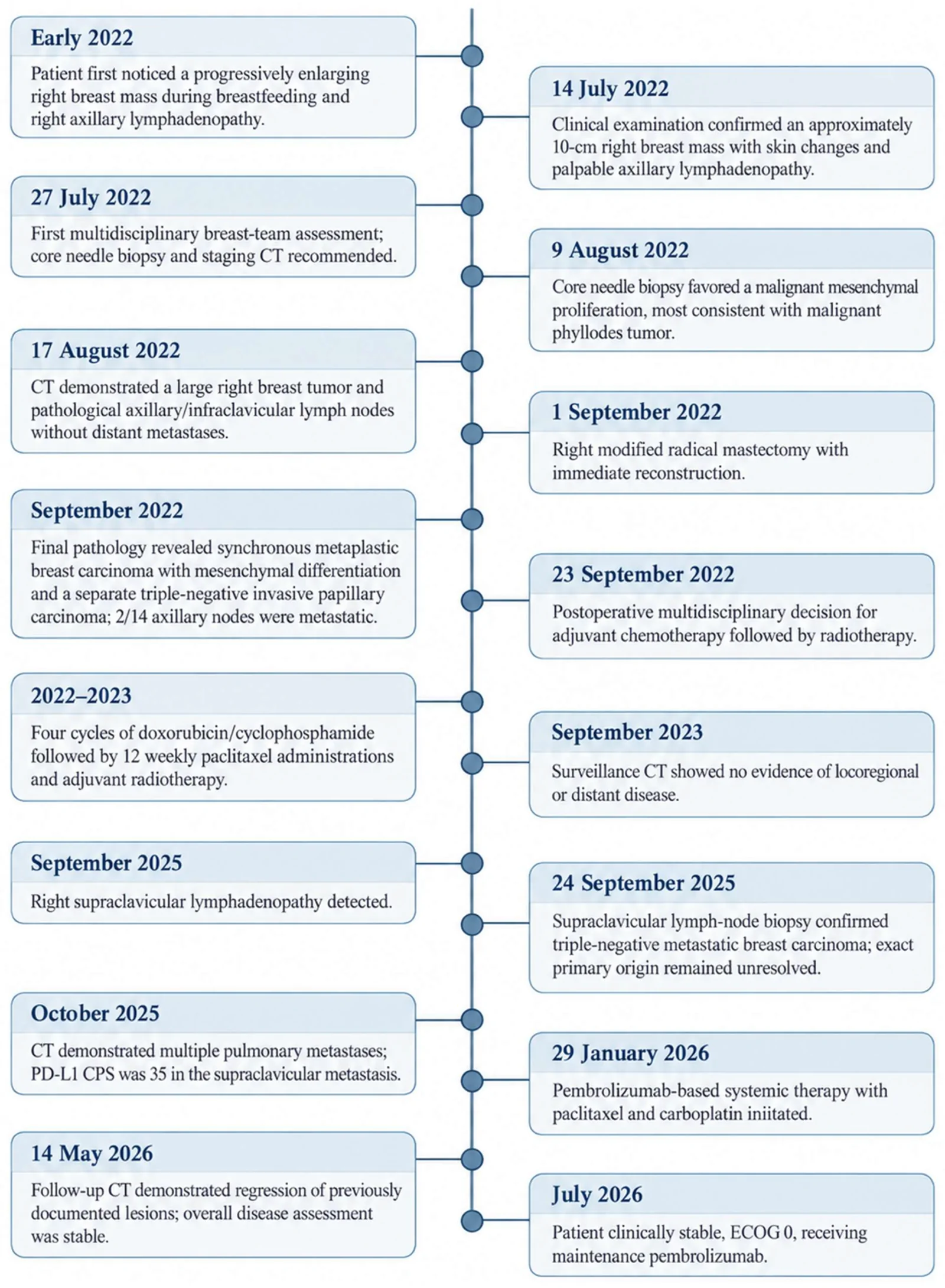
Clinical timeline of diagnosis, treatment, recurrence, and follow-up. The timeline summarizes the major clinical events from initial presentation in 2022 through diagnostic evaluation, surgery, definitive pathological diagnosis, adjuvant chemotherapy and radiotherapy, surveillance, supraclavicular recurrence and pulmonary metastatic progression, initiation of pembrolizumab-based systemic therapy, and subsequent radiological and clinical follow-up through July 2026.

At the latest documented assessment in July 2026, the patient remained clinically stable, with an Eastern Cooperative Oncology Group performance status of 0, and received the ninth cycle of maintenance pembrolizumab. Continued carboplatin administration without paclitaxel was planned at the treating center. Further computed tomography assessment of the thorax, abdomen, and pelvis was scheduled for October 2026. The overall clinical course is summarized in Figure 5.

## 3. Discussion

The principal diagnostic challenge in this case arose from the marked morphological heterogeneity of the metaplastic carcinoma. Predominance of the sarcomatoid component in the core biopsy concealed the limited epithelial component and initially led to a diagnosis of malignant phyllodes tumor. The coexistence of a separate invasive papillary carcinoma further complicated interpretation of the disease course, particularly because the initial axillary metastases were morphologically and immunophenotypically consistent with the papillary tumor. This distinction became less clear when supraclavicular recurrence and pulmonary metastases subsequently developed, as the available tissue and imaging findings were insufficient to determine which primary tumor had given rise to the later disease. In this setting, PD-L1 expression in the recurrent tumor provided a therapeutic target, and pembrolizumab-based treatment was followed by radiological regression and sustained clinical stability. The case therefore highlights the importance of adequate tumor sampling in morphologically heterogeneous breast cancers and the difficulty of determining metastatic origin when two histologically distinct primary tumors coexist.

To assess the novelty of this clinicopathological combination, we performed a targeted literature search of PubMed/MEDLINE, Scopus, Web of Science, and Google Scholar from database inception through September 2026. Search terms included combinations of ‘breast carcinosarcoma’, ‘metaplastic breast carcinoma’, ‘sarcomatoid breast carcinoma’, ‘metaplastic carcinoma with mesenchymal differentiation’, ‘invasive papillary carcinoma’, ‘papillary breast carcinoma’, ‘synchronous breast tumors’, and ‘collision tumor’, together with selected historical and overlapping terms such as ‘malignant phyllodes tumor’, ‘spindle-cell carcinoma’, and ‘biphasic breast tumor’. Reference lists of relevant publications were also manually screened. No previously published case with the same clinicopathological combination was identified.

### 3.1. Definition and Terminology

According to the current WHO classification, tumors historically described as breast carcinosarcoma are included within the spectrum of metaplastic breast carcinoma rather than recognized as a separate diagnostic entity [10]. Their classification is based on the morphological components present within the tumor. In the present case, the dominant lesion was classified as metaplastic breast carcinoma with mesenchymal differentiation. Its characteristic biphasic appearance, with clearly malignant epithelial and sarcomatoid components, corresponds to the morphology historically termed ‘carcinosarcoma’. We therefore use this term descriptively throughout the manuscript while retaining the current WHO classification as the formal diagnosis. This uncommon morphological pattern has been reported to account for approximately 0.08–0.2% of all breast malignancies [3].

The definition of invasive papillary carcinoma has also become more restrictive [11]. Earlier reports sometimes applied the term to an invasive carcinoma arising adjacent to an intraductal papillary lesion, even when the invasive component itself showed conventional non-papillary morphology [11]. Contemporary WHO criteria reserve the diagnosis for invasive adenocarcinomas in which more than 90% of the invasive component is arranged in papillary structures [11]. This stricter definition separates true invasive papillary carcinoma from conventional invasive carcinomas that merely arise in association with a papillary lesion. In the present case, more than 90% of the invasive component showed true papillary architecture with fibrovascular stromal cores. The infiltrative tumor–stroma interface and accompanying desmoplastic response further supported the diagnosis of invasive papillary carcinoma.

### 3.2. Classification of the Synchronous Breast Malignancies

Metaplastic breast carcinoma comprises a morphologically diverse group of invasive malignancies in which neoplastic epithelial cells acquire squamous, spindle-cell, or mesenchymal-appearing differentiation [3]. The current WHO framework includes low-grade adenosquamous carcinoma, fibromatosis-like metaplastic carcinoma, squamous cell carcinoma, spindle-cell carcinoma, metaplastic carcinoma with mesenchymal differentiation, and mixed metaplastic carcinoma [10]. Mesenchymal differentiation may include chondroid/chondromyxoid, osseous, rhabdomyoid, or other less common elements, depending on the morphological composition of the tumor. Tumors containing more than one metaplastic pattern are generally described as mixed metaplastic carcinomas [12]. This classification is clinically relevant because low-grade adenosquamous and fibromatosis-like tumors usually behave less aggressively than high-grade spindle-cell, squamous, mixed, and heterologous mesenchymal forms.

The position of breast carcinosarcoma has changed with the evolution of pathological terminology [8]. In the Wargotz and Norris classification, carcinosarcoma was recognized as a distinct metaplastic subtype, together with matrix-producing, spindle-cell, squamous, and osteoclastic giant-cell carcinomas [13]. In latest classifications, tumors historically designated as carcinosarcoma are encompassed within the spectrum of metaplastic carcinoma, particularly metaplastic carcinoma with mesenchymal or heterologous mesenchymal differentiation, depending on their morphological composition [8]. Nevertheless, the term remains morphologically useful when both malignant epithelial and malignant sarcomatoid components are clearly present [13]. Metaplastic carcinomas range from tumors dominated by a single metaplastic component to biphasic lesions with recognizable epithelial and sarcomatoid components [8]. Our tumor fits the biphasic category, although the marked predominance of the sarcomatous component made its epithelial origin difficult to establish on core biopsy.

The synchronous papillary malignancy belongs to a separate and equally heterogeneous group of breast tumors [14]. Malignant papillary lesions include papillary ductal carcinoma in situ, encapsulated papillary carcinoma, solid papillary carcinoma, invasive components arising in association with these lesions, and true invasive papillary carcinoma [14]. These entities differ in the distribution of myoepithelial cells, the presence of a fibrous capsule, the architecture of the epithelial proliferation, and the pattern of stromal invasion [15]. Fibrovascular cores alone are therefore insufficient for classification. The entire lesion, its interface with the surrounding breast tissue, and any associated in situ component must be assessed before a specific papillary subtype is assigned [16].

### 3.3. Incidence and Demographic Characteristics

Metaplastic breast carcinoma remains uncommon, although its recognition has increased with greater awareness of its morphological spectrum, more extensive sampling of surgical specimens, and broader use of immunohistochemistry [17,18]. Tumors reported historically as breast carcinosarcoma are considerably rarer and are represented mainly by individual case reports and small retrospective series, which limits reliable estimation of its true incidence [5]. The reported median age at diagnosis of metaplastic breast carcinoma ranges from 48 to 59 years, while large database studies have documented mean ages close to 61 years [19,20]. Some population-based analyses have reported a higher proportion of metaplastic carcinoma among African American and Hispanic women [20]. These observations remain difficult to generalize because most available cohorts are geographically restricted and may be influenced by population structure, access to specialist diagnosis, and registry coverage.

Invasive breast carcinomas with papillary features account for less than 1% of breast malignancies, but this category includes several morphologically and biologically distinct lesions [11]. True invasive papillary carcinoma has been estimated to represent approximately 13–20% of malignant papillary neoplasms and around 0.5% of invasive breast carcinomas [11,21]. Invasive papillary carcinoma predominantly affects postmenopausal women older than 50 years, with mean ages close to 60 years and median ages frequently reported in the seventh decade [11,21]. Our patient was 40 years old and premenopausal at diagnosis, placing her outside the usual demographic range described for this tumor and below the average age reported in most metaplastic carcinoma cohorts. Although invasive papillary carcinoma may coexist with other epithelial breast malignancies, its synchronous occurrence with a separate metaplastic breast carcinoma with mesenchymal differentiation appears to be exceptionally uncommon.

### 3.4. Etiology, Histogenesis, and Clonal Origin

The pathogenesis of metaplastic breast carcinoma remains incompletely understood, and no consistent precursor lesion or hereditary pathway has been established [8,22]. Individual cases have been described in association with pre-existing fibroadenoma, papilloma, spindle-cell lesions, and complex sclerosing lesions [23,24,25]. These associations suggest that metaplastic carcinoma may occasionally develop in a previously altered breast environment, but they do not establish a consistent precursor pathway [8,25]. In our patient, no previous breast lesion or clinically apparent hereditary predisposition was identified, making an apparently sporadic presentation the most appropriate interpretation.

The relationship between the epithelial and sarcomatoid components has traditionally been explained by three models [19,26]. The collision theory proposes that the two malignant populations arise independently and subsequently meet within the same tumor [19,26]. The combination theory assumes a common multipotent progenitor capable of divergent epithelial and mesenchymal differentiation [19,26]. The conversion theory proposes that the sarcomatoid component develops through metaplastic transformation of an established malignant epithelial population [19,26]. Molecular studies demonstrating shared genetic alterations in morphologically distinct components support a common clonal origin in at least a proportion of tumors [27]. These findings favor divergent differentiation or metaplastic conversion over the coincidental development of two unrelated malignancies, although no single mechanism is likely to account for every metaplastic carcinoma [19,26].

The conversion model is supported by the observation that malignant epithelial cells may progressively lose epithelial features and acquire spindle-cell morphology and mesenchymal-marker expression, a pattern consistent with epithelial–mesenchymal transition (EMT) [6,28]. During this process, malignant epithelial cells lose part of their epithelial phenotype and acquire spindle-cell morphology, mesenchymal-marker expression, and altered adhesive properties [6,28]. This may explain why cytokeratin (CK) expression is focal or absent in extensively sarcomatoid regions [6,28]. A myoepithelial pathway has also been proposed because some tumors express p63, cluster of differentiation 10 (CD10), or smooth-muscle actin (SMA), although none of these markers establishes cellular origin in isolation [29]. In our case, the predominance of a vimentin-positive sarcomatoid component with only focal epithelial-marker expression is compatible with marked metaplastic differentiation, but it does not prove a specific histogenetic mechanism. These theories apply to the epithelial and sarcomatoid components of the metaplastic carcinoma. They should not be extended automatically to the separate invasive papillary carcinoma, whose clonal relationship to the metaplastic carcinoma remains unresolved without comparative molecular analysis.

### 3.5. Molecular Landscape of the Synchronous Tumors

Metaplastic breast carcinomas frequently show basal-like or claudin-low gene-expression profiles, although they remain biologically distinct from conventional basal-like breast carcinomas [6]. Claudin-low tumors often display pronounced EMT and tumor-initiating cell characteristics, with loss of intercellular adhesion, increased invasiveness, and reduced sensitivity to apoptosis [30]. Increased activity of Snail-, BCL2-like protein 1 (BCL2L1)-, and AKT serine/threonine kinase 1 (AKT1)-associated signaling has been reported in tumors with mesenchymal differentiation [31]. Reduced activity of selected DNA-repair pathways and altered phosphatase and tensin homolog (PTEN)- and DNA topoisomerase II alpha (TOP2A)-related signaling may further contribute to their limited responsiveness to conventional chemotherapy [32,33]. These patterns are not uniform across all metaplastic subtypes, which may partly explain the substantial variation in clinical behavior and treatment response [6].

Alterations in the phosphoinositide 3-kinase (PI3K)–protein kinase B (AKT)–mammalian target of rapamycin (mTOR) pathway are among the most consistently reported molecular findings. They include activating mutations in phosphatidylinositol-4,5-bisphosphate 3-kinase catalytic subunit alpha (*PIK3CA*) and *PTEN* loss or inactivation [17]. Recurrent alterations involving tumor protein p53 (*TP53*), the telomerase reverse transcriptase (*TERT*) promoter, and the cyclin-dependent kinase inhibitor 2A/cyclin-dependent kinase inhibitor 2B (*CDKN2A/CDKN2B*) locus have also been described, although their frequency varies according to morphology [17,34]. Epidermal growth factor receptor (EGFR) overexpression and increased gene copy number occur in a proportion of tumors, but activating mutations are uncommon and receptor expression alone has not reliably predicted benefit from EGFR-directed therapy [35,36]. Aberrant Wnt/β-catenin signaling has likewise been reported, while the variable frequency of catenin beta 1 (*CTNNB1*) mutations indicates that pathway activation may arise through several mechanisms [37,38]. These findings identify potentially relevant biological vulnerabilities, but none currently defines a molecularly uniform subgroup or a standard targeted treatment for metaplastic breast carcinoma with mesenchymal differentiation [17].

Papillary breast lesions also show considerable molecular diversity despite their shared architecture [39]. *AKT1* and *PIK3CA* alterations have been identified in papillomas without atypia, papillomas with usual ductal hyperplasia or atypical ductal hyperplasia; lesions associated with ductal carcinoma in situ and malignant papillary tumors [40]. Their presence across this spectrum suggests that PI3K–AKT activation may participate in the development of papillary growth but is not sufficient to determine invasion or aggressive behavior [41]. Neuroblastoma RAS viral oncogene homolog (*NRAS*) mutations have been reported in smaller subsets, while increasing chromosomal complexity and loss of heterozygosity may accompany progression [40,41]. Alterations involving chromosome 16 have been found in both proliferative papillomas and papillary carcinomas, whereas additional abnormalities involving chromosomes 3, 7, 17, and X appear more closely associated with malignant disease [41,42]. Several signaling pathways, including c-Met, rearranged during transfection/papillary thyroid carcinoma (RET/PTC), integrin, Sonic Hedgehog (SHH), and bone morphogenetic protein (BMP) signaling have also been proposed in papillary morphogenesis, but their precise contribution remains uncertain [39]. Papillary architecture should therefore be regarded as a neoplasia-associated growth pattern rather than a marker of one specific molecular pathway [39].

Molecular studies of papillary carcinomas have largely examined conventional papillary tumors, with available cohorts dominated by encapsulated, solid, and other low- to intermediate-grade, hormone receptor-positive lesions [43]. When matched for grade and receptor status, these lesions may share several mutations with invasive breast carcinoma of no special type while showing fewer copy-number abnormalities and lower expression of genes involved in proliferation and cellular motility [44,45]. These observations fit the generally indolent behavior of many conventional papillary carcinomas but cannot be applied directly to every invasive papillary tumor [11,43]. The papillary carcinoma in our patient was high grade, highly proliferative, and associated with lymphovascular invasion and axillary metastases, placing it outside the profile represented in most genomic series [43]. No genomic profiling was performed on either synchronous tumor or on the recurrent supraclavicular metastasis; therefore, no specific driver alteration can be assigned, no molecular relationship between the two primary tumors can be inferred, and the clonal origin of the later recurrent disease cannot be established [46]. Broader sequencing could become clinically relevant in metastatic disease, particularly if additional treatment options are required after immunotherapy-based management [47].

### 3.6. Clinical Presentation of the Synchronous Tumors

Metaplastic breast carcinoma most commonly presents as a palpable mass and may initially be clinically indistinguishable from conventional invasive carcinoma [48,49]. A more suggestive feature is rapid enlargement over a relatively short interval, a presentation particularly well documented in high-grade spindle-cell and carcinosarcomatous tumors [48]. Pain is variably reported and is not a defining presenting feature, although it may accompany larger or locally advanced lesions [50]. Clinical presentation therefore appears to be driven largely by rapid tumor growth and local burden rather than by a subtype-specific symptom pattern [48,50]. Tumor dimensions vary widely among published cohorts [48]. Some institutional series have reported mean diameters close to 3 cm, whereas cohorts enriched in advanced disease have included much larger lesions. In one study, the mean diameter was 7.4 cm and 42% of patients presented with T4 disease [48].

Continued expansion may lead to erythema, skin fixation, ulceration, nipple distortion, or prominent superficial vessels [51,52]. These findings indicate extensive local involvement but do not distinguish metaplastic carcinoma from malignant phyllodes tumor, primary breast sarcoma, or another rapidly growing high-grade malignancy [53]. Clinical–pathological correlation is therefore essential, particularly when a bulky lesion has a relatively circumscribed or otherwise nonspecific appearance [54,55]. In our patient, the sequence of self-detection followed by progressive enlargement, extensive breast involvement, erythema, and fixation of the overlying skin closely matched the locally advanced clinical pattern described for metaplastic breast carcinoma with mesenchymal differentiation showing carcinosarcoma morphology.

Invasive papillary carcinoma also most often presents as a palpable breast mass [11]. Bloody nipple discharge has been reported in approximately one third of patients and probably reflects the frequent relationship between papillary tumors and the ductal system [11,56]. Subareolar location and multifocality may occur, but neither feature is sufficiently specific to separate invasive papillary carcinoma from benign papillary lesions or other breast carcinomas [11]. The clinical contribution of the papillary tumor in our case could not be distinguished from that of the dominant metaplastic breast carcinoma. The much larger metaplastic tumor determined the palpable abnormality and local skin changes, whereas the separate 22-mm papillary carcinoma remained clinically concealed within the overall tumor burden. Its biological relevance became apparent only after histological examination demonstrated papillary differentiation in both the breast lesion and the initial axillary metastases.

### 3.7. Radiological Appearance and Limitations of Imaging in Synchronous Breast Malignancies

Breast carcinosarcoma has no pathognomonic radiological appearance [57]. Its imaging features overlap with those of conventional invasive carcinoma, malignant phyllodes tumor, primary breast sarcoma, and other metaplastic carcinomas [57]. Some lesions are irregular and infiltrative, whereas others are round, oval, lobulated, or relatively well circumscribed [57,58]. A sharply defined contour may therefore underestimate the biological aggressiveness of the tumor [58]. Imaging is more reliable for defining local extent, identifying secondary tissue changes, and assessing operability than for establishing the precise histological subtype [54,59].

On ultrasonography, metaplastic carcinoma most often appears as a hypoechoic or heterogeneous mass with variable shape and margins [60,61]. Posterior acoustic enhancement may occur instead of the shadowing more commonly associated with conventional invasive carcinoma [58,60]. Complex solid–cystic architecture is frequent, and the cystic areas may correspond to necrosis, hemorrhage, cystic degeneration, or myxoid change [60]. Internal vascularity is often concentrated in the viable solid or peripheral portions [62,63]. Invasive papillary carcinoma may produce a similar sonographic pattern, appearing either as a hypoechoic solid lesion or as a complex cystic–solid mass with septations, anechoic hemorrhagic spaces, posterior enhancement, and internal Doppler flow [11,64]. These shared features limit lesion-specific interpretation when both malignancies occur in the same breast [60]. In our patient, ultrasonography demonstrated a large hypoechoic hypervascular tumor together with adjacent cystic lesions, but the preoperative examination could not reliably assign individual radiological findings to the 125-mm metaplastic breast carcinoma or the separate 22-mm papillary carcinoma.

On mammography, metaplastic carcinoma usually presents as a high-density mass with circumscribed, partially circumscribed, indistinct, or irregular margins [65]. Calcifications are absent in most cases but have been described in up to one quarter of tumors, particularly in matrix-producing carcinomas with chondroid or osseous differentiation [66]. Invasive papillary carcinoma may also appear as an oval or lobulated high-density mass, sometimes accompanied by microcalcifications [11]. More aggressive papillary-pattern carcinomas may instead show irregular shape, indistinct borders, or speculation [67,68]. Neither a circumscribed outline nor the absence of calcifications is sufficiently reassuring when the clinical examination indicates rapid progression [69]. Clinicoradiological discordance should prompt tissue diagnosis or reassessment rather than continued imaging surveillance [69].

Magnetic resonance imaging of metaplastic carcinoma commonly shows heterogeneous T2 hyperintensity and heterogeneous or peripheral ring enhancement [70]. T2-hyperintense areas may correspond to necrosis, hemorrhage, myxoid matrix, cystic degeneration, or edematous stroma [70,71]. Enhancement kinetics varies and may show type II or type III curves, although less suspicious patterns have occasionally been reported [70]. Malignant papillary lesions can also display high T2 signal, peripheral enhancement, rapid enhancement followed by washout, or non-mass enhancement with an internally clustered-ring pattern [72]. These findings may support suspicion of malignancy but do not reliably distinguish invasive papillary carcinoma from benign papilloma, ductal carcinoma in situ with papillary architecture, or another invasive breast cancer [72,73]. Morphological and kinetic findings must therefore be interpreted together with ultrasound and tissue sampling [73].

Computed tomography is particularly useful in large tumors because it defines the relationship to the skin, pectoral fascia, and chest-wall musculature and may demonstrate extensive necrotic or hemorrhagic change [74,75]. In our patient, CT confirmed that the dominant tumor occupied almost the entire breast, involved the nipple, and closely approached the pectoral fascia without unequivocal muscular invasion. No distant disease was identified at the initial staging examination. These findings guided surgical planning but did not resolve the distinction between metaplastic breast carcinoma with mesenchymal differentiation and malignant phyllodes tumor or reveal the full pathological significance of the smaller papillary carcinoma. The case therefore shows an additional limitation of imaging in synchronous disease: the largest lesion may dominate the radiological picture and obscure a smaller tumor with independent metastatic potential.

### 3.8. Pathological Features and Limitations of Preoperative Sampling

Cytological recognition of tumor historically described as breast carcinosarcoma depends on the simultaneous identification of malignant epithelial and mesenchymal-appearing cells [76,77]. When only one population is aspirated, the lesion may be interpreted as a poorly differentiated carcinoma, primary sarcoma, or another high-grade spindle-cell neoplasm [77,78]. Marked pleomorphism establishes malignancy but does not demonstrate the biphasic nature of the tumor [77]. Cytology may therefore suggest a metaplastic process, but reliable subclassification usually requires preservation of tissue architecture and examination of the relationship between the different cellular components [79].

Core biopsy provides more architectural information but remains vulnerable to sampling error in large and heterogeneous tumors [25,80]. In tumors historically described as breast carcinosarcoma, the epithelial and sarcomatoid components may differ markedly in proportion and may occupy spatially separate areas [55,57]. A core composed predominantly of pleomorphic spindle cells can therefore resemble primary breast sarcoma or the malignant stromal component of a phyllodes tumor [25,81]. Immunohistochemistry does not always resolve this problem because epithelial-marker expression may be focal and absent from the sampled area [82]. In our patient, core biopsy showed a highly pleomorphic, vimentin-positive proliferation without AE1/AE3 expression. In the absence of an identifiable epithelial component, interpretation as a malignant mesenchymal neoplasm favoring malignant phyllodes tumor was consistent with the sampled morphology rather than contradictory to the definitive diagnosis.

At gross examination, metaplastic breast carcinoma with mesenchymal differentiation showing carcinosarcoma morphology often forms a large, fleshy or firm mass with areas of hemorrhage, necrosis, and cystic degeneration [57]. The margins may appear relatively distinct despite aggressive biological behavior [5,57]. This combination may further suggest malignant phyllodes tumor, particularly when the lesion is bulky and the sarcomatoid component predominates [83]. Grossly different regions should therefore be sampled separately, including solid, hemorrhagic, necrotic, peripheral, and transitional areas [3]. In the present case, the dominant tumor measured 125 mm and showed extensive necrotic and hemorrhagic change. Its large size and marked internal heterogeneity emphasized the need for broad sampling of the mastectomy specimen.

Histologically, tumors historically described as breast carcinosarcoma are characterized by the coexistence of an unequivocally malignant epithelial component and a malignant sarcomatoid or mesenchymal-appearing component [3]. The epithelial areas may show ductal, glandular, or squamous differentiation, whereas the sarcomatoid areas may consist of spindle-shaped, pleomorphic, or multinucleated malignant cells [3,55]. Heterologous elements, including chondroid, osseous, rhabdomyoblastic, or other forms of mesenchymal differentiation, may also be present [3]. The two components may be closely intermingled, connected by transitional areas, or separated by a more distinct interface. In our patient, approximately 95% of the tumor consisted of high-grade pleomorphic sarcomatoid tissue, while poorly differentiated epithelial sheets represented only a small component. This pronounced imbalance explains why the epithelial differentiation was not identified in the limited preoperative sample.

The second malignancy required a different morphological assessment. Papillary differentiation is defined by true fibrovascular stromal cores covered by neoplastic epithelial cells, but papillary architecture alone does not establish a specific diagnosis [14,84]. The pathologist must also demonstrate genuine stromal invasion and distinguish invasive papillary carcinoma from papillary ductal carcinoma in situ, encapsulated papillary carcinoma, solid papillary carcinoma, and conventional invasive carcinoma associated with a papillary lesion [85]. The tumor–stroma interface, distribution of myoepithelial cells, presence of desmoplasia, associated in situ disease, and pattern of invasion should all be considered [14]. In our case, the smaller tumor showed a predominantly papillary invasive growth pattern with true fibrovascular cores and an infiltrative interface with the surrounding desmoplastic stroma. Lymphatic and vascular invasion, together with morphologically concordant papillary carcinoma in the axillary lymph nodes, provided additional evidence of its invasive and metastatic behavior.

Papillary architecture also requires specific attention during histological grading [39]. Papillae should not be counted as tubular differentiation because they do not reproduce the normal terminal duct–lobular unit [39]. Many invasive papillary carcinomas therefore receive a tubule-formation score of 3, while the final Nottingham grade depends on the combined assessment of architecture, nuclear pleomorphism, and mitotic activity [39,86]. The papillary carcinoma in our patient was classified as histological grade 3. It measured 22 mm and contained extensive necrotic and hemorrhagic areas, features that further distinguished it from the more commonly reported low-grade papillary tumors.

The coexistence of two distinct malignancies substantially increased the risk of preoperative underdiagnosis [80]. The core biopsy sampled the dominant sarcomatoid population but did not capture the small epithelial component required for the diagnosis of carcinosarcoma or the separate papillary carcinoma. The definitive diagnosis became possible only after extensive examination of the mastectomy specimen. This case therefore illustrates two simultaneous forms of sampling error: underrepresentation of a minor epithelial component within a heterogeneous biphasic tumor and complete omission of a smaller synchronous malignancy.

### 3.9. Immunohistochemical Profile and Biological Phenotype of the Synchronous Tumors

Immunohistochemistry is particularly valuable when metaplastic breast carcinoma is dominated by spindle-cell, pleomorphic, or mesenchymal-appearing tissue [25,87]. Broad-spectrum cytokeratins, epithelial membrane antigen, and CK7 may reveal epithelial differentiation that is inconspicuous on routine sections and help distinguish metaplastic carcinoma from a primary breast sarcoma [88,89]. Staining may nevertheless be focal and confined to only a few tumor cells [25]. A negative result with a single cytokeratin antibody therefore does not exclude metaplastic carcinoma, especially in a limited core biopsy [25]. Vimentin is commonly expressed in sarcomatoid areas, but its broad distribution among mesenchymal and sarcomatoid neoplasms makes it supportive rather than diagnostic [89]. In our patient, the core biopsy contained a vimentin-positive, AE1/AE3-negative malignant proliferation, whereas examination of the resection specimen identified focal epithelial-marker expression and a small morphologically recognizable carcinomatous component. This combination established the biphasic nature of the tumor and supported the diagnosis of metaplastic breast carcinoma with mesenchymal differentiation showing carcinosarcoma morphology.

Additional antibodies should be selected according to the suspected line of differentiation. Desmin, myogenin, and myogenic differentiation 1 (MyoD1) may support rhabdomyoblastic differentiation; S100 and SRY-box transcription factor 10 (SOX10) may assist in evaluating selected neural, myoepithelial, or cartilaginous patterns; and special AT-rich sequence-binding protein 2 (SATB2) may be useful when osteoid production is present [90,91]. Cluster of differentiation 31 (CD31) and ETS-related gene (ERG) are more reliable than cluster of differentiation 34 (CD34) for confirming endothelial differentiation because CD34 also highlights stromal cells and intratumoral vessels [92]. Expression of p63, SMA, or CD10 may occur in metaplastic carcinoma but does not independently establish myoepithelial origin [87,93]. In the present carcinosarcoma, focal p63 expression and positivity for S100, cluster of differentiation 68 (CD68), and SMA characterized the sarcomatoid population but did not, in the absence of corresponding morphological differentiation, define a specific heterologous lineage. Immunohistochemical findings were therefore interpreted in the context of the biphasic morphology rather than as isolated evidence of tumor origin.

Most metaplastic breast carcinomas lack estrogen receptor (ER), progesterone receptor (PR), and human epidermal growth factor receptor 2 (HER2) expression, with a triple-negative phenotype reported in approximately 77–89% of cases [48,94]. Receptor status should be confirmed by testing rather than assumed from the tumor morphology [95]. This is particularly important in synchronous tumors, because separate primary malignancies may show different therapeutic profiles [96]. In our patient, both the metaplastic breast carcinoma and the invasive papillary carcinoma were ER-negative, PR-negative, and HER2-negative. Their proliferative activity was also high but differed quantitatively: the Ki-67 index was approximately 60% in the metaplastic breast carcinoma and 40% in the invasive papillary carcinoma. These findings supported the high-grade biological phenotype of both lesions while confirming that endocrine and HER2-directed treatments were not applicable to either primary tumor.

This immunophenotype was particularly unusual for the papillary carcinoma. Conventional invasive papillary carcinomas are predominantly hormone receptor-positive, HER2-negative tumors and usually show lower proliferative activity [11,97]. The papillary tumor in our patient instead combined grade 3 morphology with cytokeratin 7 (CK7) and cytokeratin 5/6 (CK5/6) expression, absence of ER, PR, and HER2, and a Ki-67 index of approximately 40%. CK5/6 positivity demonstrated basal cytokeratin expression but should not be regarded as proof of a molecularly defined basal-like subtype [98]. The initial axillary metastases retained papillary morphology, were CK7-positive and cytokeratin 20 (CK20)-negative, and remained triple-negative, with an increased Ki-67 index of approximately 50%. These findings supported their origin from the invasive papillary carcinoma. They also showed that papillary architecture in this case was not associated with the indolent phenotype described in predominantly low-grade, hormone receptor-positive papillary carcinoma cohorts. Instead, histological grade, proliferation, lymphovascular invasion, and nodal spread more accurately reflected its biological behavior.

Immune biomarkers provided additional clinically relevant information after metastatic progression. PD-L1 expression has been reported in a substantial subgroup of metaplastic breast carcinomas, although prevalence varies according to the antibody clone, scoring method, tissue compartment, and morphological subtype [99]. In our patient, PD-L1 testing with the 22C3 antibody showed a CPS of 25 in both synchronous primary tumors—the metaplastic breast carcinoma with mesenchymal differentiation and the invasive papillary carcinoma—and a CPS of 35 in the supraclavicular metastasis. The supraclavicular lesion was classified as metastatic breast carcinoma of no special type, expressed E-cadherin, remained ER-negative, PR-negative, and HER2-negative, and showed a Ki-67 index of approximately 40%. Its exact relationship to either of the two synchronous primary tumors could not be established from the available morphology and immunophenotype.

### 3.10. Differential Diagnosis of the Synchronous Breast Tumors

The differential diagnosis of tumors historically described as breast carcinosarcoma includes malignant phyllodes tumor, primary breast sarcoma, spindle-cell metaplastic carcinoma, matrix-producing carcinoma, myoepithelial carcinoma, and selected benign or reactive spindle-cell proliferations [57,87]. These lesions may all contain highly cellular spindle-shaped or pleomorphic areas with marked nuclear atypia, brisk mitotic activity, necrosis, and hemorrhage [83,100]. The degree of cytological atypia is therefore less discriminating than the overall architecture, the nature of the epithelial component, and its relationship to the sarcomatoid tissue [83]. Demonstration of unequivocally malignant epithelial differentiation within a predominantly sarcomatoid tumor is central to the diagnosis of carcinosarcoma [5].

Malignant phyllodes tumor is the most important alternative diagnosis in a large biphasic breast neoplasm dominated by malignant stromal-appearing tissue [53,87]. It typically retains a fibroepithelial architecture characterized by elongated epithelial-lined clefts, leaf-like projections, subepithelial stromal condensation, and variable stromal overgrowth [83]. Its epithelial component is usually benign, although an independent carcinoma may occasionally arise within a phyllodes tumor [83]. In carcinosarcoma, both the epithelial and sarcomatoid components are malignant, and the epithelial areas may be intermingled with, directly continuous with, or transition into the sarcomatoid population [13]. In the present case, the absence of recognizable malignant epithelial differentiation in the core biopsy initially favored malignant phyllodes tumor, whereas the epithelial component identified in the resection specimen supported the diagnosis of carcinosarcoma.

Primary breast sarcomas may show a similarly high-grade spindle-cell or pleomorphic morphology but lack a malignant epithelial component [87]. The relevant alternatives include undifferentiated pleomorphic sarcoma, fibrosarcoma, osteosarcoma, angiosarcoma, and other uncommon lineage-defined sarcomas [101]. Matrix-producing carcinoma should be considered when chondromyxoid, cartilaginous, or osseous material is present, but it is characterized by a direct transition from carcinoma to extracellular matrix without an intervening spindle-cell component [102]. Spindle-cell metaplastic carcinoma may be almost entirely monophasic and can require extensive examination to identify focal epithelial differentiation [25]. Myoepithelial carcinoma may express cytokeratins, p63, smooth-muscle actin, S100, and related markers, but this combination is not specific without corresponding morphological features [29]. Fibromatosis, scar tissue, and other reactive spindle-cell lesions generally lack severe atypia and unequivocal malignant epithelial differentiation, although deceptively bland areas may occasionally occur in metaplastic carcinoma [87,103].

The differential diagnosis of the synchronous papillary tumor includes intraductal papilloma, papillary ductal carcinoma in situ, encapsulated papillary carcinoma, solid papillary carcinoma, and invasive carcinoma associated with a papillary lesion [39,85]. Although these lesions share papillary or fibrovascular architecture to varying degrees, they differ in the distribution of myoepithelial cells, the configuration of the tumor boundary, and the presence and pattern of stromal invasion [14,39]. Encapsulated papillary carcinoma is usually surrounded by a fibrous capsule and has a circumscribed growth pattern, whereas solid papillary carcinoma is dominated by compact cellular nodules traversed by delicate fibrovascular networks [14,104]. Genuine invasive papillary carcinoma is characterized by malignant epithelial cells arranged around true fibrovascular cores within an infiltrative stromal growth pattern; metastatic disease, when present, provides additional evidence of its invasive nature [39]. In our patient, the presence of recognizable fibrovascular papillae, lymphatic and vascular invasion, and morphologically corresponding papillary deposits in two axillary lymph nodes supported the invasive and metastatic nature of the papillary malignancy.

Invasive papillary carcinoma must also be distinguished from invasive micropapillary carcinoma [39]. The latter lacks true fibrovascular cores and instead forms cohesive epithelial clusters within clear stromal spaces, often with reversed apical polarity [105,106]. This distinction is clinically important because invasive micropapillary carcinoma has a marked tendency toward lymphovascular invasion and regional nodal spread [106]. Despite the presence of these aggressive features in our case, the breast tumor and nodal metastases retained genuine papillary structures with fibrovascular stromal cores, arguing against a micropapillary pattern [11,39]. Tall cell carcinoma with reversed polarity may also enter the differential diagnosis of a CK5/6-positive papillary-pattern tumor [107,108]. However, the characteristic combination of tall columnar cells, reversed nuclear polarity, and solid-papillary architecture, often accompanied by thyroid-like nuclear grooves or pseudoinclusions and occasionally follicle-like structures, was not identified in the present lesion. CK5/6 expression alone is therefore insufficient for reclassification and should be interpreted together with the architectural findings [107].

### 3.11. Patterns of Distant Metastatic Spread

Metaplastic breast carcinoma has a pronounced tendency toward hematogenous dissemination, with the lungs consistently reported as the most frequent site of distant disease [109]. In one cohort, pulmonary metastases developed in 22% of patients, followed by bone metastases in 13%, brain metastases in 6%, and liver metastases in 4%, while splenic and adrenal involvement was uncommon [110]. Studies focused specifically on tumors historically described as breast carcinosarcoma have described a similar distribution, with the lungs and pleura accounting for a substantial proportion of distant treatment failures [5,111]. Pulmonary surveillance is therefore particularly relevant in patients with large, high-grade metaplastic tumors, although no individual pathological feature can reliably predict the timing or anatomical distribution of progression [50].

Distant metastasis appears uncommon in conventional invasive papillary carcinoma series, although these estimates should be interpreted cautiously because many cohorts are dominated by older patients with hormone receptor-positive, lower-grade, and earlier-stage disease [112]. Papillary architecture may also change during tumor progression and may be reduced or completely lost in a metastatic deposit [39]. Conversely, a metastatic carcinoma may develop papillary-like structures that were not prominent in the primary tumor [39]. The origin of a distant metastasis in a patient with synchronous breast malignancies should therefore not be determined from architecture or metastatic site alone [39].

In our patient, the initial staging examination showed no distant metastases, and subsequent surveillance imaging in September 2023 remained negative for recurrent or metastatic disease. Multiple pulmonary metastases were identified on computed tomography in October 2025, demonstrating progression from initially localized disease to visceral dissemination. The pulmonary lesions were identified radiologically and were not separately sampled; their origin therefore cannot be assigned with certainty to either the metaplastic breast carcinoma or the invasive papillary carcinoma. Although the thoracic distribution is compatible with the recognized metastatic pattern of metaplastic carcinoma, anatomical concordance alone is insufficient to establish lineage in a patient with two biologically aggressive primary tumors. This uncertainty should be preserved in the interpretation of the disease course rather than resolved on the basis of the expected metastatic behavior of either histological subtype.

### 3.12. Regional Lymphatic Spread and Origin of Nodal Metastases

Axillary lymph-node metastases occur less consistently in metaplastic breast carcinoma than in conventional invasive breast carcinoma [61,113]. Reported frequencies vary widely, from very low rates in small series or selected subgroups to approximately 40–50% in some cohorts enriched in locally advanced disease; older reports have described rates as high as 56% [58,114,115]. Several studies have documented axillary involvement in approximately one third of patients, while a National Cancer Database analysis of 329 patients with tumors historically described as breast carcinosarcoma found nodal disease in approximately 23% of cases [111,113]. This variation reflects differences in tumor stage, cohort size, pathological classification, and the proportion of individual metaplastic subtypes. The relatively modest average rate of nodal involvement should not, however, be interpreted as evidence that regional staging can be omitted, because metaplastic carcinomas are capable of both lymphatic and hematogenous dissemination [116].

Clinical or radiological lymph-node enlargement does not reliably establish metastatic involvement [117]. Reactive hyperplasia and inflammation may produce nodal enlargement or suspicious morphology, while necrotic changes require particular caution because they may also occur in metastatic nodes [118]. Histological assessment therefore remains necessary even when the primary lesion has a recognized tendency toward blood-borne spread [119]. Conversely, a clinically unremarkable axilla does not exclude microscopic nodal disease [117]. Regional staging remains relevant for prognosis and treatment planning, although the frequent combination of a bulky metaplastic primary tumor and limited nodal involvement supports a greater relative tendency toward hematogenous dissemination in this tumor group [116].

Axillary lymph-node metastases are reported less frequently in invasive papillary carcinoma than in invasive breast carcinoma of no special type [11]. Since axillary nodal involvement is less common in conventional invasive papillary carcinoma, its presence may indicate more aggressive tumor behavior rather than simply reflecting the papillary growth pattern [120]. In our patient, two of 14 axillary lymph nodes contained metastatic carcinoma with papillary architecture. The deposits retained CK7 expression, lacked CK20, and showed the same triple-negative phenotype as the synchronous papillary primary, with a Ki-67 index of approximately 50%. Together with the preserved fibrovascular papillary structures, these findings strongly supported their relationship to the 22-mm invasive papillary carcinoma rather than to the much larger metaplastic carcinoma. Importantly, this pathological attribution should be distinguished from formal nodal staging: the pN category reflects the regional nodal status of the overall ipsilateral breast cancer presentation and was therefore recorded at the case level rather than separately for each synchronous primary tumor.

The later supraclavicular recurrence was more difficult to assign. Unlike the initial axillary deposits, the biopsy was described as a solid, moderately differentiated breast carcinoma of no special type and did not reproduce the previously documented papillary architecture. Papillary differentiation may be reduced or lost during metastatic progression because of intratumoral heterogeneity, clonal selection, or limited sampling of the recurrent lesion [39]. Architecture alone is therefore insufficient to establish the source of a metastasis in a patient with two synchronous primary tumors [39]. Comparison of cytological features and immunophenotype may narrow the possibilities, but definitive attribution may require comparative molecular analysis. In the present case, the supraclavicular metastasis remained triple-negative and expressed E-cadherin, but these findings were not specific enough to link it conclusively to either the invasive papillary carcinoma or the metaplastic breast carcinoma. Its origin should therefore remain unresolved in the manuscript. Definitive attribution would have required comparative molecular profiling of the synchronous primary tumors and the recurrent lesion, which was not performed.

### 3.13. Multidisciplinary Management

The management of metaplastic breast carcinoma with mesenchymal differentiation requires close integration of clinical, radiological, and pathological findings [5]. Imaging may accurately define the extent of disease while remaining unable to determine its histological subtype, and core biopsy may represent only one region of a markedly heterogeneous tumor [121]. A diagnosis based on limited tissue should therefore be reconsidered when it does not fully account for the clinical or radiological presentation [121]. Communication among the radiologist, breast surgeon, and pathologist is particularly important when a rapidly enlarging mass shows predominantly spindle-cell or mesenchymal morphology [87]. In such circumstances, radiological–pathological correlation, targeted sampling of morphologically distinct areas, and extensive examination of the resection specimen may substantially alter the final classification. In our patient, this integrated approach explained why the initial biopsy favored malignant phyllodes tumor while the mastectomy specimen established a metaplastic breast carcinoma with mesenchymal differentiation showing carcinosarcoma morphology and a separate invasive papillary carcinoma.

When two distinct breast malignancies coexist, treatment planning should incorporate the independent pathological contribution of each tumor rather than rely solely on the size or apparently more aggressive morphology of the dominant lesion [122,123]. The 125-mm grade 3 metaplastic breast carcinoma represented the major local disease burden and determined the complexity of surgical excision and reconstruction. The smaller papillary carcinoma was nevertheless clinically consequential because it was also grade 3, showed lymphatic and vascular invasion, and its morphological and immunohistochemical profile strongly supported its relationship to the metastases identified in two axillary lymph nodes. The pathological stage and postoperative risk profile therefore reflected the combined adverse features of both tumors. This distinction is important because a smaller or conventionally more favorable histological subtype should not be disregarded when it independently contributes to nodal stage or systemic risk. In the present case, the combination of a very large high-grade metaplastic breast carcinoma, a biologically aggressive papillary carcinoma, lymphovascular invasion, and regional nodal involvement supported adjuvant anthracycline- and taxane-based chemotherapy followed by irradiation of the chest wall and regional lymphatic areas.

Multidisciplinary assessment remained necessary during follow-up [124]. The development of a supraclavicular recurrence together with pulmonary metastases prompted repeat pathological evaluation of the accessible recurrent lesion, reassessment of receptor status, and additional biomarker testing [125]. Because the recurrent nodal carcinoma could not be conclusively assigned to either primary tumor, treatment decisions were based on the phenotype and extent of the current metastatic disease rather than on an unproven assumption regarding its origin [126]. PD-L1 testing subsequently identified a clinically actionable biomarker and supported eligibility for pembrolizumab-based systemic treatment [127].

### 3.14. Neoadjuvant Systemic Therapy and Rationale for Upfront Surgery

Neoadjuvant systemic therapy is routinely considered in locally advanced, triple-negative, and HER2-positive breast cancer when tumor downstaging may facilitate resection, increase the likelihood of breast conservation, or provide prognostic information through assessment of pathological response [128]. Metaplastic breast carcinoma, however, is generally less sensitive to conventional chemotherapy than non-metaplastic breast cancer with a similar receptor profile [129,130]. Reported pathological complete response rates are generally low, ranging from 0% in some small series to approximately 23% in prospective data, with substantial variability across cohorts [48,131,132]. In one retrospective cohort, only 7.4% of patients achieved a pathological complete response, whereas 25.9% experienced progression during treatment [48]. These findings indicate that the response expected in conventional triple-negative breast cancer cannot be applied directly to metaplastic carcinoma.

Individual series further illustrate the limited and heterogeneous activity of conventional regimens [133]. Chen et al. reported partial responses in 18.2% of patients treated in the neoadjuvant setting and in 8.3% of those receiving first-line chemotherapy [133]. No objective responses were observed with several anthracycline-, cyclophosphamide-, or vinorelbine-based regimens, although occasional responses occurred with taxane-containing treatment [133]. In an observational comparison, the response rate to anthracycline- and taxane-based neoadjuvant chemotherapy was 12.5% in metaplastic carcinoma compared with 75% in non-metaplastic triple-negative breast cancer, and none of the metaplastic tumors achieved a complete response [134]. Neoadjuvant therapy may therefore remain appropriate for initially unresectable disease or when meaningful downstaging is required, but its use should be individualized and accompanied by close clinical and radiological reassessment, because chemoresistant tumors may continue to progress while definitive local treatment is delayed [135].

Evidence for neoadjuvant treatment of invasive papillary carcinoma is even more limited [11]. Available IPC cohorts are dominated by relatively small, early-stage and hormone receptor-positive tumors, while IPC-specific prospective evidence for neoadjuvant therapy is essentially absent [97]. For clinically significant high-grade or receptor-negative true invasive papillary carcinomas, treatment decisions are therefore generally extrapolated from evidence for conventional invasive breast cancer with the corresponding stage and biological phenotype [11]. Neoadjuvant chemotherapy may be considered when downstaging is necessary, but papillary morphology alone does not constitute an indication [128]. Diagnostic certainty is also important because papillary breast lesions encompass several entities with markedly different invasive potential and systemic treatment requirements [10]. In a patient with more than one breast malignancy, limited preoperative sampling may fail to identify the tumor that ultimately determines systemic risk.

In our patient, neoadjuvant treatment could have been considered on the basis of the initial clinical stage and extensive local disease. However, staging computed tomography showed no distant metastases or unequivocal invasion of the pectoral muscle, and the tumor was considered surgically resectable. The core biopsy favored a malignant mesenchymal neoplasm, most likely malignant phyllodes tumor, and did not establish either metaplastic breast carcinoma with mesenchymal differentiation or invasive papillary carcinoma. In this preoperative context, upfront surgery provided prompt local control and was consistent with the working diagnosis. Extensive examination of the mastectomy specimen subsequently revealed a large carcinosarcoma and a separate high-grade invasive papillary carcinoma whose morphology and immunophenotype closely matched those of the axillary nodal metastases. The indication for systemic chemotherapy therefore became clear only after definitive pathological assessment. Given the uncertain chemosensitivity of metaplastic carcinoma and the absence of a preoperative diagnosis of the papillary malignancy, the choice of primary surgery should be regarded as a reasonable multidisciplinary decision rather than a missed opportunity for neoadjuvant treatment.

### 3.15. Surgical Management of the Synchronous Breast Tumors

Surgery remains the principal treatment for resectable metaplastic breast carcinoma with mesenchymal differentiation [5]. The operative approach should be determined by tumor size, local extension, breast-to-tumor ratio, skin or chest-wall involvement, and the possibility of obtaining microscopically negative margins [136]. Histological subtype alone does not mandate mastectomy. Although mastectomy has been reported in approximately 36–92% of patients with metaplastic breast carcinoma, this wide range largely reflects differences in stage at presentation, tumor volume, referral patterns, and the feasibility of breast preservation [48,136]. Retrospective comparisons have not consistently demonstrated a survival advantage for mastectomy over breast-conserving surgery [136]. Conservation followed by radiotherapy may therefore be appropriate for selected localized tumors when complete excision with acceptable margins and cosmetic outcome is feasible [136,137].

The situation differs when a large metaplastic breast carcinoma with mesenchymal differentiation produces an unfavorable tumor-to-breast volume ratio or extensive skin and soft-tissue involvement [57]. In such cases, breast-conserving surgery may be technically unsuitable because adequate margins and acceptable residual breast volume may be difficult to achieve, even when imaging suggests a relatively circumscribed mass [136]. Axillary management should be guided by the clinical and pathological nodal findings rather than by the generally lower average frequency of nodal metastasis reported for metaplastic carcinoma [116]. In our patient, the dominant tumor occupied almost the entire breast and was associated with skin fixation and clinically abnormal regional lymph nodes. A right modified radical mastectomy therefore offered the most realistic possibility of complete local excision and simultaneously allowed definitive axillary staging. Microscopically negative margins were achieved, while examination of the axillary specimen showed papillary morphology and an immunophenotypic profile strongly supporting a relationship between the nodal metastases and the separate invasive papillary carcinoma rather than the metaplastic breast carcinoma.

Surgical treatment of invasive papillary carcinoma otherwise follows the principles applied to other invasive breast carcinomas [11,97]. Breast-conserving surgery is appropriate when the invasive disease can be completely removed with clear margins and an acceptable breast-conserving result, whereas mastectomy is generally reserved for extensive or multicentric disease, an unfavorable tumor-to-breast ratio, inability to achieve negative margins, or other anatomical or treatment-related contraindications to breast conservation [138]. Sentinel lymph-node biopsy remains appropriate for most clinically node-negative invasive papillary carcinomas, while the need for more extensive axillary treatment is determined by clinical and pathological nodal status and the overall treatment setting [11]. Papillary morphology alone should not lead to a less intensive surgical approach. Tumor size, invasive extent, grade, lymphovascular invasion, and nodal involvement remain the relevant determinants [139]. In the present case, mastectomy was required primarily because of the 125-mm metaplastic breast carcinoma and the extent of local disease, but the 22-mm papillary carcinoma was independently important because it showed lymphatic and vascular invasion and produced metastases in two axillary lymph nodes. The operation therefore addressed the combined burden of two malignant processes rather than a single dominant tumor alone.

Evidence concerning immediate reconstruction in metaplastic breast carcinoma is limited and strongly affected by patient selection [48,140]. Apparent differences in recurrence among reconstructed and non-reconstructed patients should not be interpreted as evidence that reconstruction modifies tumor biology [48,141]. The decision depends on the anticipated tissue defect, margin requirements, planned radiotherapy, wound-healing risk, comorbidities, and patient preference [142]. In our patient, reconstruction was required primarily to cover the extensive skin and soft-tissue defect created by oncologically adequate resection. A transposition flap combined with a Wolfe full-thickness skin graft provided definitive coverage, although marginal ischemic change and limited dry necrosis developed during postoperative recovery. This outcome illustrates that reconstruction can facilitate radical excision in locally advanced disease, but it also introduces wound-healing considerations that must be incorporated into subsequent systemic and radiation-treatment planning.

### 3.16. Adjuvant Systemic Therapy

No adjuvant regimen has been established specifically for metaplastic breast carcinoma with mesenchymal differentiation [5]. Treatment generally follows the principles used for high-risk metaplastic breast carcinoma and, because these tumors are often triple-negative, may also incorporate systemic regimens used for triple-negative invasive breast cancer [3,5]. This treatment strategy is not supported by prospective studies specific to metaplastic breast carcinoma with mesenchymal differentiation. Most available evidence comes from retrospective series that include heterogeneous metaplastic subtypes, disease stages, and treatment regimens [5,48]. The benefit of adjuvant treatment is therefore difficult to quantify. Nevertheless, adjuvant chemotherapy is commonly considered for patients with high-risk disease, particularly in the presence of large tumor size, high histological grade, nodal involvement, or a triple-negative phenotype, because these features collectively indicate a substantial risk of recurrence [48,143].

The relatively low chemosensitivity of metaplastic carcinoma does not imply that systemic treatment is uniformly ineffective [144,145,146]. Some retrospective analyses have suggested better survival among patients who received chemotherapy in addition to surgery [144]. Kennedy et al., for example, reported a higher five-year overall survival with combined systemic treatment than with surgery alone [111]. Such observations should be interpreted cautiously because retrospective treatment comparisons remain vulnerable to treatment-selection bias and residual confounding, even after adjustment for measured clinicopathological variables [111]. Even so, these data do not support routine omission of chemotherapy solely because the tumor belongs to the metaplastic spectrum [144].

Anthracycline- and taxane-containing regimens are used most often, while older cyclophosphamide, methotrexate, and 5-fluorouracil (CMF)-based combinations appear to have less consistent activity [26,147]. Hennessy et al. reported unfavorable outcomes with CMF, whereas patients receiving anthracycline-based treatment appeared to fare better [148]. Sarcoma-oriented regimens have also been used in small numbers of patients with dominant mesenchymal differentiation, but the available experience is insufficient to show that they are superior to standard breast-cancer chemotherapy [147]. Likewise, there is currently insufficient evidence to support selecting systemic therapy according to a specific metaplastic component, including the use of platinum-based regimens for tumors with prominent squamous differentiation [115]. Morphology may suggest biological differences between subtypes, but it cannot yet determine a validated chemotherapy regimen [149].

Therapeutic decisions should therefore integrate stage, tumor burden, grade, nodal status, receptor profile, proliferative activity, and the patient’s general condition [139]. The frequent triple-negative phenotype supports the use of chemotherapy but should not create an expectation of the same treatment sensitivity seen in conventional triple-negative breast carcinoma [129,150]. In our patient, the combination of extensive high-grade disease, regional nodal involvement, and absence of endocrine or HER2-directed treatment options supported postoperative doxorubicin and cyclophosphamide followed by weekly paclitaxel. This regimen was in line with current treatment approaches for high-risk triple-negative breast cancer, but there is no carcinosarcoma-specific evidence establishing it as a standard of care [126,139].

Evidence for adjuvant chemotherapy in invasive papillary carcinoma is also limited because published cohorts are dominated by small, low-grade, hormone receptor-positive tumors [151,152]. Chemotherapy should not be prescribed solely because papillary carcinoma is invasive, nor should papillary morphology be interpreted as evidence that systemic treatment is unnecessary [151]. The decision should instead incorporate invasive tumor size, Nottingham grade, receptor profile, proliferative activity, lymphovascular invasion, and regional nodal status [120,151]. Small, low-grade, node-negative papillary carcinomas may derive little additional benefit from chemotherapy, whereas high-grade, receptor-negative, lymphovascular invasion-positive, or node-positive tumors represent a substantially different clinical setting [151]. The generally favorable outcomes reported for conventional papillary carcinoma therefore cannot be applied directly to biologically aggressive papillary tumors.

#### HER2-Positive Metaplastic Breast Carcinoma

Although metaplastic breast carcinoma is predominantly triple-negative, receptor-defined subgroups do not appear to have identical clinical outcomes. A Surveillance, Epidemiology, and End Results (SEER)-based analysis of 1,516 patients identified HER2 positivity in only 5.2% of tumors, compared with a triple-negative phenotype in 64.1% [153]. Despite its small size, the HER2-positive subgroup had a three-year overall survival of 91.8%, compared with 75.4% for triple-negative and 77.1% for hormone receptor-positive/HER2-negative disease [153]. After multivariable adjustment, survival did not differ significantly between HER2-positive metaplastic carcinoma and HER2-positive invasive ductal carcinoma [153]. These findings support the possibility that access to HER2-directed therapy may partly offset the adverse biological features associated with metaplastic histology. However, the registry-based design, limited subgroup size, and incomplete information on individual treatment regimens prevent a direct attribution of the survival difference to anti-HER2 therapy. HER2 testing should therefore be performed in every metaplastic carcinoma rather than assuming triple negativity from morphology alone; in our patient, the absence of HER2 expression excluded this therapeutic pathway [154].

### 3.17. Role of Endocrine Therapy

Endocrine therapy has a limited role in metaplastic breast carcinoma with mesenchymal differentiation because most tumors lack estrogen and progesterone receptor expression [5]. The low frequency of hormone-receptor positivity may partly reflect the limited conventional glandular differentiation present in many metaplastic tumors, although morphology alone does not reliably predict hormone-receptor status [155]. Endocrine treatment should therefore be considered only when estrogen or progesterone receptor expression is clearly documented in the invasive component [95,156]. This distinction is particularly important in morphologically heterogeneous or synchronous tumors, in which separate malignant components may not necessarily share the same receptor profile [157]. In our patient, the reported absence of estrogen and progesterone receptor expression provided no rationale for endocrine therapy, leaving chemotherapy and radiotherapy as the available postoperative systemic and locoregional treatment options.

### 3.18. EGFR as a Potential Therapeutic Target

Epidermal growth factor receptor (EGFR) overexpression has been reported in approximately 70–80% of metaplastic breast carcinomas in classic series, although the reported frequency varies across cohorts. This has made EGFR one of the more frequently investigated potential therapeutic targets in this disease [158]. Increased receptor expression or gene copy number may support proliferative and prosurvival signaling, particularly in tumors with basal-like or mesenchymal features [32]. However, immunohistochemical overexpression does not necessarily indicate oncogenic dependence on the EGFR pathway. Activating EGFR mutations appear to be rare, while the relationships among receptor expression, gene amplification, pathway dependence, and treatment response remain incompletely defined [159]. EGFR status should therefore be interpreted as a potential biomarker for further investigation rather than as evidence that an EGFR inhibitor will be clinically effective [35].

Clinical experience with EGFR-directed therapy is limited to small series and individual reports [149]. Outside the metaplastic setting, Sabatier et al. reported a marked but temporary response to cetuximab in a heavily pretreated patient with EGFR-amplified metastatic triple-negative breast cancer [160]. Together, these findings provide a biological rationale for EGFR targeting in selected molecularly defined tumors, but they do not establish the efficacy of cetuximab or small-molecule EGFR inhibitors across the broader metaplastic population. The distinction between receptor overexpression and genomic amplification may also be clinically relevant, although it has not been prospectively validated [35,158]. EGFR-directed treatment should therefore remain investigational and, when considered in advanced disease, should preferably be guided by comprehensive molecular profiling or offered within a clinical trial [149]. EGFR expression and amplification were not assessed in our patient, so no case-specific therapeutic implication can be drawn from the reported prevalence alone.

### 3.19. Immunotherapy and Immune Checkpoint Inhibition

Immune checkpoint inhibition has expanded the treatment options for selected patients with triple-negative breast cancer, but evidence specific to metaplastic breast carcinoma remains limited [161,162]. Interest in this approach is supported by the relatively frequent expression of programmed death-ligand 1 (PD-L1) reported in some metaplastic tumors, together with molecular features associated with immune evasion [99,163]. Epithelial–mesenchymal transition (EMT) and activation of the phosphoinositide 3-kinase (PI3K)/protein kinase B (AKT) signaling pathway have both been linked to increased PD-L1 expression [164]. However, these molecular features vary among metaplastic carcinomas and do not necessarily indicate an immune-responsive tumor phenotype or predict sensitivity to immune checkpoint inhibition.

Responses to pembrolizumab and atezolizumab have been reported in individual patients and small series with advanced metaplastic breast carcinoma [94,165]. Some of these responses were clinically meaningful, including in tumors that had progressed after conventional chemotherapy [166,167]. The published experience nevertheless remains too limited and heterogeneous to define a treatment strategy specific to carcinosarcoma or to individual metaplastic subtypes. Patients differed in histology, treatment line, PD-L1 status, accompanying chemotherapy, and response assessment. These reports therefore support the possibility of therapeutic benefit in selected cases but do not show that checkpoint inhibition can overcome the broader chemoresistant phenotype of metaplastic carcinoma. Importantly, this histology-specific evidence is considerably weaker than the randomized evidence supporting pembrolizumab-based therapy in PD-L1-positive metastatic triple-negative breast cancer and should not be regarded as equivalent.

PD-L1 also has an uncertain prognostic meaning in this setting [99]. Increased expression has been associated with unfavorable outcomes in some retrospective studies, possibly reflecting aggressive tumor biology and an immunosuppressive microenvironment [168]. At the same time, it may identify a subgroup with greater potential sensitivity to immune checkpoint blockade [127]. This apparent dual role makes individual testing more informative than assumptions based on histological subtype alone [99].

From a future-treatment perspective, PD-L1 expression may remain clinically relevant if the disease progresses, particularly when a new metastatic site is accessible for biopsy. Because biomarker expression may vary between primary and metastatic sites and may evolve during treatment, reassessment of PD-L1 in recurrent or progressive disease may provide more relevant information than relying on the original primary-tumor result alone. Persistent or increased PD-L1 expression could support consideration of immune-checkpoint-based strategies or eligibility for clinical trials evaluating novel immunotherapy combinations. However, PD-L1 positivity alone should not be interpreted as evidence that continuation or rechallenge with pembrolizumab after progression will be beneficial, and subsequent treatment selection should incorporate prior exposure, pattern of progression, toxicity, receptor status, and additional actionable molecular findings [125,126,127].

In the present case, the rationale for pembrolizumab-based treatment was derived primarily from evidence for PD-L1-positive metastatic triple-negative breast cancer rather than from evidence specific to either of the two synchronous histological subtypes. The phase III KEYNOTE-355 trial established a survival benefit for pembrolizumab combined with chemotherapy in previously untreated locally recurrent unresectable or metastatic triple-negative breast cancer with a PD-L1 combined positive score (CPS) of at least 10 [131]. The biopsied supraclavicular recurrence in our patient retained a triple-negative phenotype and showed a PD-L1 CPS of 35, providing a biomarker-based rationale for immune checkpoint inhibition. However, evidence specifically supporting pembrolizumab in metaplastic breast carcinoma remains limited to small series and individual reports, and no histology-specific standard has been established. This distinction is particularly relevant because the supraclavicular recurrence could not be conclusively attributed to either the metaplastic carcinoma or the invasive papillary carcinoma, while the pulmonary lesions were not biopsied. Therefore, the observed radiological regression and sustained clinical stability should be interpreted as disease control achieved with a pembrolizumab-containing regimen in PD-L1-positive metastatic triple-negative breast cancer rather than as evidence of treatment efficacy against one particular histological subtype.

### 3.20. Emerging Molecularly Targeted Therapies

Frequent alterations in the PI3K–AKT–mammalian target of rapamycin (mTOR) signaling pathway have prompted investigation of combination therapies targeting this pathway in advanced metaplastic breast carcinoma [169]. In a phase I study of 52 patients, temsirolimus or everolimus was combined with bevacizumab and liposomal doxorubicin to inhibit intracellular proliferative signaling, tumor angiogenesis, and cellular proliferation simultaneously [170]. The objective response rate was 21%, including complete responses in 8% and partial responses in 13% of patients [170]. A further 19% maintained stable disease for at least six months, producing a clinical benefit rate of 40% [170]. These results do not indicate broad sensitivity across the metaplastic population, but they show that a subset of heavily treated patients may achieve meaningful disease control with a pathway-directed combination.

Molecular analysis was available for 43 tumors, of which 32 (74%) contained at least one activating aberration in the PI3K pathway; phosphatidylinositol-4,5-bisphosphate 3-kinase catalytic subunit alpha (PIK3CA) mutation was the most frequent individual alteration, detected in 19 patients [170]. Objective responses were more frequent in tumors with a detected PI3K-pathway aberration than in those without one, at 31% and 0%, respectively [170]. This difference suggested that PI3K-pathway alterations might have predictive value for objective tumor response. However, the clinical benefit rate was almost identical in the two groups, at 44% and 45% [170]. In this small cohort, detectable PI3K-pathway aberrations were associated with objective tumor shrinkage but did not clearly identify patients more likely to achieve prolonged disease control [170]. Interpretation is further limited by the small sample and the use of an assay restricted to selected hotspot mutations Some tumors classified as having no detected PI3K-pathway aberration may nevertheless have harbored non-hotspot PIK3CA variants, unrecognized PTEN loss, or alterations in other components of the pathway [170]. Broader genomic profiling may provide a more accurate measure of pathway dependence than hotspot testing alone.

Individual clinical responses also support molecular selection, although they cannot define a treatment standard [171]. A patient with PIK3CA H1047R-mutated metastatic metaplastic carcinoma who had been enrolled in the BELLE-4 trial achieved a confirmed partial response lasting approximately 17 months with paclitaxel plus the pan-PI3K inhibitor buparlisib, with an overall survival of 42 months after the development of metastatic disease [171]. The case suggests that matching a dominant molecular alteration with a pathway-directed agent may occasionally produce durable benefit. At present, however, such treatment should be considered investigational and most relevant in unresectable, recurrent, or metastatic disease after comprehensive molecular assessment [47]. These molecularly targeted strategies were not used in our patient. Because comprehensive genomic profiling was not performed on the recurrent disease, no molecularly selected targeted therapy could be considered. Such profiling may become relevant if further disease progression occurs and standard systemic treatment options become limited.

### 3.21. Biological Behavior and Prognostic Determinants of the Synchronous Breast Tumors

Metaplastic breast carcinoma is generally associated with less favorable outcomes than conventional invasive breast carcinoma [50]. Population-based analyses support this difference [172,173]. In one study including 1,011 patients with metaplastic carcinoma and more than 250,000 patients with invasive ductal carcinoma, five-year survival was 78% and 93%, respectively [172]. In a separate institutional comparison, five-year overall survival was 54.5% for metaplastic carcinoma, 85.1% for invasive ductal carcinoma, and 73.3% for triple-negative invasive ductal carcinoma [174]. The poorer outcome of metaplastic carcinoma therefore cannot be explained by receptor status alone [172]. High histological grade, marked intratumoral heterogeneity, early hematogenous dissemination, and limited sensitivity to conventional systemic therapy probably contribute to its distinctive clinical course [50].

Reported survival estimates nevertheless vary considerably. Across retrospective studies, five-year overall survival has ranged from approximately 50% to 83%, while five-year disease-free survival has generally ranged from 41% to 65% [174,175,176]. In breast carcinosarcoma cohorts, a median overall survival of 8.7 years and a five-year overall survival of approximately 60% have been reported [111]. These differences reflect variation in stage distribution, pathological terminology, morphological subtype, treatment, and duration of follow-up. Older series may also include tumors that would now be assigned to different diagnostic categories. Published estimates should therefore be interpreted as broad risk indicators rather than precise predictions for an individual patient.

The metaplastic spectrum itself contains tumors with substantially different behavior [4]. Low-grade adenosquamous and fibromatosis-like metaplastic carcinomas generally follow a more indolent course, although incomplete excision may lead to local recurrence [177,178]. High-grade spindle-cell, squamous, mixed, and mesenchymally differentiated tumors are more frequently associated with early recurrence and distant progression [4]. Even within the high-grade group, the independent prognostic value of morphology remains inconsistent across studies [179,180]. Prognostic assessment therefore continues to rely primarily on established clinicopathological variables, including tumor size, T category, skin involvement, histological grade, lymphovascular invasion, regional nodal status, resection margins, and the presence of distant disease [111,181]. Extensive necrosis, high proliferative activity, and limited response to systemic treatment may provide additional information, but no individual immunohistochemical or molecular marker has been validated as a stand-alone prognostic tool for metaplastic breast carcinoma with mesenchymal differentiation [129].

Invasive breast carcinomas with papillary features are usually associated with favorable outcomes, with some retrospective series reporting five-year disease-free or relative survival rates approaching 90–95% [11,39]. These figures are strongly influenced by the predominance of low-stage, low-grade, hormone receptor-positive, and circumscribed papillary tumors in published cohorts. They should not be applied uniformly to high-grade invasive papillary carcinomas. For encapsulated or solid papillary lesions with a distinct, unequivocal invasive component, pathological T staging should be based on the largest invasive focus rather than on the total extent of the associated circumscribed papillary proliferation [16]. Prognosis is more reliably determined by invasive tumor size, Nottingham grade, lymphovascular invasion, nodal status, receptor profile, and adequacy of locoregional treatment than by papillary architecture itself [39].

Metaplastic breast carcinoma with mesenchymal differentiation carries a substantial risk of treatment failure, with available series showing that recurrences are concentrated particularly within the first two to three years after definitive treatment [48]. Published series have reported highly variable disease-free or recurrence-free survival, with approximately one-half of patients remaining free of recurrence at five years in several cohorts, although estimates vary substantially according to stage, histological composition, treatment, and duration of follow-up [148,182]. These estimates should be interpreted cautiously because the available studies are small and differ in stage distribution, pathological classification, treatment, and duration of observation. Nevertheless, they consistently indicate that a period of initial disease control does not eliminate the risk of subsequent locoregional or distant progression.

Follow-up should address both the operative region and the potential sites of systemic dissemination [183]. Clinical examination of the chest wall and regional lymphatic basins remains important, while new respiratory, skeletal, neurological, or constitutional symptoms should prompt appropriate investigation [183]. In synchronous disease, surveillance should not be based solely on the expected behavior of the larger or histologically more aggressive tumor. In our patient, regional and pulmonary progression after an initial interval without detectable disease reinforced this principle. Because the recurrent disease could not be conclusively attributed to either primary tumor, follow-up should address the overall disease burden rather than the anticipated behavior of a single histological component.

Several limitations of this case should be acknowledged. First, as a single-patient report, the observations cannot be generalized to the broader population of metaplastic or papillary breast carcinomas. Second, comparative molecular profiling of the two synchronous primary tumors and the later supraclavicular recurrence was not performed, precluding definitive assessment of their clonal relationships and the origin of the recurrent disease. Third, the pulmonary metastases were diagnosed radiologically and were not biopsied. Finally, the clinical response observed after metastatic progression occurred during a multi-agent pembrolizumab-containing regimen and therefore cannot be attributed either to pembrolizumab alone or to one particular histological subtype. These limitations should be considered when interpreting the diagnostic and therapeutic implications of the present case.

Long-term management should remain adaptive to the evolving pattern of disease. In patients with persistent metastatic disease, serial clinical and radiological reassessment is necessary to document the duration of disease control, treatment tolerance, and the emergence of new metastatic sites. When progression occurs at an accessible site, repeat biopsy may be useful to reassess receptor status and clinically actionable biomarkers, particularly when the biological phenotype of the recurrent disease cannot be reliably inferred from the original primary tumors. In such circumstances, broader molecular profiling may also become relevant when standard systemic options are exhausted or when eligibility for biomarker-driven clinical trials is being considered.

## 4. Conclusions

Metaplastic breast carcinoma with mesenchymal differentiation and invasive papillary carcinoma are individually uncommon tumors, and their synchronous occurrence creates substantial diagnostic and therapeutic complexity. The present case illustrates the importance of extensive and representative sampling, careful morphological and immunohistochemical characterization of each tumor, and direct comparison of metastatic deposits with all synchronous primary lesions. Although the initial axillary metastases were most consistent with the invasive papillary carcinoma, the origin of the later supraclavicular recurrence and pulmonary metastatic disease remained unresolved, and the absence of comparative molecular profiling precluded definitive assessment of clonal relationships. The observed disease control during pembrolizumab-containing therapy should therefore be interpreted in the context of PD-L1-positive metastatic triple-negative breast cancer and not as evidence of pembrolizumab efficacy against either metaplastic breast carcinoma or invasive papillary carcinoma specifically. More broadly, the available literature supports a multidisciplinary approach in which treatment decisions are guided by stage, biological phenotype, metastatic pattern, and reassessment of clinically actionable biomarkers at disease progression rather than by histological designation alone.

## Data Availability

This article, being a case report and literature review, does not contain original primary data. The information presented is derived from previously published sources and the patient’s medical records.

## Abbreviations

The following abbreviations are used in this manuscript:

AE1/AE3	Pancytokeratin AE1/AE3
AKT	Protein kinase B
AKT1	AKT serine/threonine kinase 1
BCL2L1	BCL2-like protein 1
BMP	Bone morphogenetic protein
CD10	Cluster of differentiation 10
CD31	Cluster of differentiation 31
CD34	Cluster of differentiation 34
CD68	Cluster of differentiation 68
CDKN2A	Cyclin-dependent kinase inhibitor 2A
CDKN2B	Cyclin-dependent kinase inhibitor 2B
CK	Cytokeratin
CK5/6	Cytokeratin 5/6
CK7	Cytokeratin 7
CK20	Cytokeratin 20
CMF	Cyclophosphamide, methotrexate, and 5-fluorouracil
CT	Computed tomography
CTNNB1	Catenin beta 1
DNA	Deoxyribonucleic acid
EGFR	Epidermal growth factor receptor
EMT	Epithelial–mesenchymal transition
ER	Estrogen receptor
ERG	ETS-related gene
HER2	Human epidermal growth factor receptor 2
Ki-67	Ki-67 proliferation marker
mTOR	Mammalian target of rapamycin
MyoD1	Myogenic differentiation 1
NRAS	Neuroblastoma RAS viral oncogene homolog
p63	Tumor protein p63
PD-L1	Programmed death-ligand 1
PI3K	Phosphoinositide 3-kinase
PIK3CA	Phosphatidylinositol-4,5-bisphosphate 3-kinase catalytic subunit alpha
PR	Progesterone receptor
PTEN	Phosphatase and tensin homolog
RET/PTC	Rearranged during transfection/papillary thyroid carcinoma
S100	S100 calcium-binding protein
SATB2	Special AT-rich sequence-binding protein 2
SEER	Surveillance, Epidemiology, and End Results
SHH	Sonic Hedgehog
SMA	Smooth-muscle actin
SOX10	SRY-box transcription factor 10
TERT	Telomerase reverse transcriptase
TOP2A	DNA topoisomerase II alpha
TP53	Tumor protein p53
WHO	World Health Organization
